# Anks1a regulates COPII-mediated anterograde transport of receptor tyrosine kinases critical for tumorigenesis

**DOI:** 10.1038/ncomms12799

**Published:** 2016-09-13

**Authors:** Haeryung Lee, Hyuna Noh, Jiyoung Mun, Changkyu Gu, Sanja Sever, Soochul Park

**Affiliations:** 1 Department of Biological Science, Sookmyung Women's University, Chungpa-ro 47gil 100, Yongsan-gu, Seoul 140-742, Korea; 2 Department of Biomedical Laboratory Science, College of Health Science, Eulji University, Seongnam-Si, Gyeonggi-Do 13135, Korea; 3 Division of Nephrology, Massachusetts General Hospital, Charlestown, Massachusetts 02129, USA

## Abstract

ErbB2 signalling, which is amplified by EphA2 binding, is an important therapeutic target for breast cancer. Despite the importance of the EphA2/ErbB2 complex in promoting breast tumorigenesis, the mechanism by which these receptor tyrosine kinases (RTKs) are exported from the endoplasmic reticulum (ER) remains poorly understood. Here we report that the PTB adaptor Anks1a is specifically localized to the ER on its own serine phosphorylation. Once there, Anks1a acts as an important regulator of COPII-mediated EphA2 ER export. The Anks1a ankyrin repeat domain binds EphA2 and causes it to accumulate at sites of ER exit. Simultaneously, the Anks1a PTB domain binds Sec23. This induces internalization of EphA2 via COPII vesicles, while Anks1a remains behind on the ER membrane. EphA2 also binds ErbB2 in the ER and seems to load ErbB2 into growing COPII carriers. Together, our study reveals a novel mechanism that regulates the loading of RTKs into COPII vesicles.

## Abstract (web summary)

EphA2/ErbB2 complex is important in promoting breast cancer but the mechanism by which these receptor tyrosine kinases are exported from the endoplasmic reticulum is unknown. Here the authors show that Anks1a acts as a cargo adaptor in sorting EphA2 into COPII vesicles, thus modulating the surface level of EphA2.

## Graphical abstract

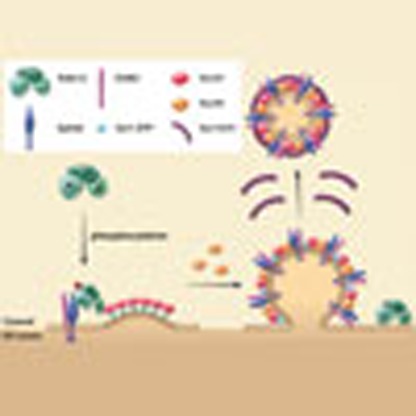

Cells must sense and respond to a dynamically changing environment. The receptor tyrosine kinases (RTKs) are a large family of cell surface receptors that detect various extracellular growth factors, cytokines and hormones. As such, RTKs are key regulators of normal cellular signalling and their dysfunction is associated with many types of cancer^1^,^2^. The function of these transmembrane receptors depends on their proper trafficking from the intracellular membranes of the endoplasmic reticulum (ER) to the plasma membrane at the surface^3^. Cells use several strategies to control the trafficking of newly synthesized RTKs to the cell surface. Some RTKs may be retained inactive in the ER and eventually degraded if they are not needed. If a cell requires high levels of RTK on the plasma membrane, it may direct the ER membranes to rapidly release mature RTKs and send them towards the cell surface. This anterograde trafficking from the ER to the plasma membrane first sees proteins sent to the Golgi via COPII vesicles. The precise mechanisms that direct the selective sorting of RTKs into specific COPII vesicles for exit from the ER are not well understood, but there are clearly other proteins involved.

The Anks1 family of adaptor proteins is a subgroup of phosphotyrosine-binding domain (PTB) adaptors with two members, Anks1a and Anks1b (refs 4, 5, 6, 7). Both contain six ankyrin repeats (ANK), two sterile alpha motif (SAM) domains and a PTB. They are known to function as signalling adaptors downstream of RTKs such as the EGF and Eph receptors^5^,^6^,^8^,^9^. Recently, Anks1a was shown to increase EGF-induced EGFR internalization from the plasma membrane into recycling endosomes while blocking its routing to the lysosome^10^. Another PTB adaptor, Munc18-interacting protein (Mint), is important for the export of amyloid precursor protein from the Golgi towards LAMP1-positive structures^11^,^12^,^13^,^14^. It seems reasonable, therefore, that the Dab-like PTB adaptors may share a general function in modulating transmembrane protein trafficking throughout the endomembrane system^7^. Sorting of transmembrane proteins in and out of each endomembrane compartment may begin with the recruitment of a specific PTB adaptor from the cytosol. This would require specific cytoplasmic motifs in the transmembrane cargos for binding to distinct PTB adaptors. These interactions would, in turn, facilitate the loading of each cargo into a coated carrier via interactions with the proteins responsible for carrier biogenesis. Each mature carrier would then have targeting information specifying a distinct membrane compartment and for interacting with the additional machinery involved in uncoating and fusion with that compartment.

EphA2 RTK is overexpressed in many different types of cancer cell lines and cancer stem cells^15^,^16^,^17^,^18^ and elevated EphA2 correlates with advanced tumour staging, more rapid disease progression and lower patient survival. This is interesting because physiological activation of EphA2 by ephrins is known to inhibit tumorigenesis. Evidence suggests that when EphA2 is overexpressed, it promotes cell proliferation and invasiveness without engaging an ephrin ligand. Overexpression of EphA2 activates Ras/Erk signalling, which then downregulates ephrin ligands^19^,^20^. This leads to a pathological imbalance between the EphA2 receptor and ephrin ligands that may be important for promoting the ligand-independent oncogenic potential of EphA2 in various cancer cell types. In addition, growth factor receptors are known to enhance the ephrin-independent oncogenic function of EphA2. In particular, the physical association between EphA2 and ErbB2 enhances Ras/Erk signalling^21^. This EphA2-mediated amplification of ErbB2 signalling contributes to breast tumour initiation and metastasis in mouse mammary tumour virus (MMTV)-ErbB2/Neu transgenic mice. Since the ER must supply the mature forms of EphA2 and ErbB2 required for the synergistic and oncogenic signalling of these receptors at the cell surface, the precise mechanism by which these receptors are effectively exported from the ER in cancer cells is an important unresolved question. In this study, we identify a novel role for the Anks1a PTB adaptor in the ER for the selective sorting of the EphA2 and ErbB2 RTK cargos into COPII-coated vesicles.

## Results

### Anks1a interacts with EphA2 in the ER to regulate its export

**Figure 1:**
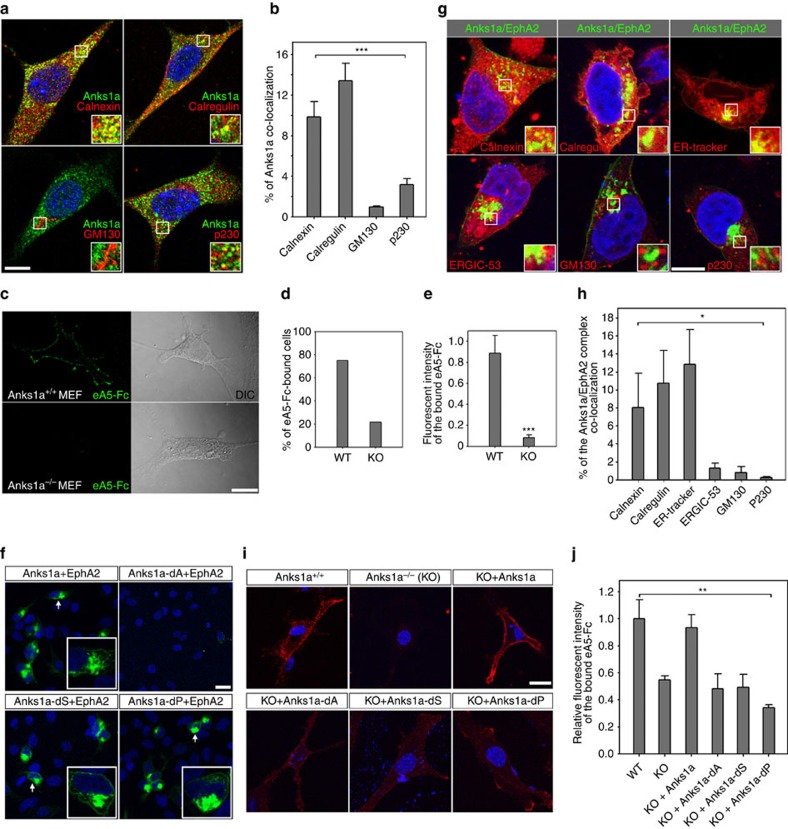
Interaction of Anks1a with EphA2 is involved in the transport of EphA2 from the ER to the plasma membrane. (**a**) CT26 cells were stained with an anti-Anks1a antibody. Subcellular organelles were detected with ER markers (Calnexin and Calregulin) or Golgi markers (GM130 and p230). Scale bar, 10 μm. (**b**) Anks1a co-localization with the ER or Golgi was calculated using Imaris (*n*=15 for each antibody). ***P<0.001, one-way analysis of variance (ANOVA) test. (**c**) MEF cells from wild-type or Anks1a-null mutant mice were incubated with 1 μg ml^*−*1^ ephrinA5-Fc at 4 °C for 1 h and ephrinA5-Fc at the surface was visualized via fluorescein isothiocyanate (FITC)-conjugated anti-human IgG. Scale bar, 25 μm. (**d**) The percentage of ephrinA5-Fc-bound cells (*n*=25 for both WT and knockout (KO) MEFs). (**e**) Total fluorescence of cell-surface-bound ephrinA5-Fc was calculated using ImageJ (*n*=25 for both WT and KO MEFs). Data represent means±s.e. ***P<0.001, Student's *t*-test. (**f**) VC-tagged EphA2 was co-transfected into HEK293T cells with VN-tagged Anks1a constructs. Cells were fixed and analysed for BiFC signals. Scale bar, 20 μm. (**g**) HEK293T cells were transfected with both VN-tagged Anks1a and VC-tagged EphA2 before being stained with the indicated subcellular markers. Scale bar, 10 μm. (**h**) Co-localization efficiency for the Anks1a/EphA2 complex with each marker was calculated using Imaris (*n*=20 for each transfection). *P<0.05, one-way ANOVA test. (**i**) Anks1a KO MEF cells were transfected with the indicated Anks1a constructs. Surface-binding assays were performed 48 h after transfection as described in **c**. The transfected cells were detected by an anti-GFP antibody that recognizes the VN portion fused to each Anks1a protein. Scale bar, 20 μm. (**j**) Fluorescent intensity was calculated as described in **b** (*n*=15 for each transfection). **P<0.01, one-way ANOVA test. Data represent means±s.e.

Although Anks1a has been implicated in EphA receptor-mediated signalling, its precise role remains unknown^5^,^6^. Since CT26 (colon carcinoma derived) cells endogenously express high levels of both Anks1a and EphA2, we used this cell line for studying the subcellular localization of Anks1a. The Anks1a expressed in CT26 cells appears in a punctate pattern that co-localizes with the ER markers Calnexin and Calregulin, but not the Golgi markers GM130 and p230 (Fig. 1a,b). Anks1a is also co-localized with Sec16, a bona fide marker for the ER exit site (ERES), at levels similar to that observed with other ER markers (Supplementary Fig. 1a,b). Anks1a's co-localization with other markers, such as those specific for endosomes and lysosomes, is ∼10-fold lower (Supplementary Fig. 1a,b). To explore the potential role of Anks1a in the ER, we cultured primary mouse embryonic fibroblasts (MEFs) from *Anks1a* wild-type (WT) or null-mutant embryos at E14.5. MEF cells express both Anks1a and EphA2 at moderate levels^5^. To identify EphA2 localized to the plasma membrane, we used ephrinA5-Fc fusion protein and visualized their interactions at the cell surface. We found that the fraction of ephrinA5-bound cells is 3.5-fold lower in *Anks1a* knockout MEFs than controls (Fig. 1c,d). *Anks1a* mutant MEF cells exhibit a 9-fold lower fluorescence intensity than that of control MEF cells (Fig. 1e).

To determine whether EphA2 and Anks1a interact with one another, we used bimolecular fluorescence complementation (BiFC)^22^ in 293 cells. We added the N- (VN) and C-terminal (VC) fragments of YFP to Anks1a and EphA2, respectively. In the BiFC technique, reconstitution of YFP fluorescence suggests a protein–protein interaction. Control transfections of EphA2-VC with VN or Anks1a-VN with VC do not produce any fluorescence (Supplementary Fig. 1c). In contrast, we detected a fluorescent signal at the plasma membrane and within vesicular structures in the cell, indicating the presence of Anks1a/EphA2 complexes (Fig. 1f, first panel). To identify the domain responsible for these Anks1a–EphA2 interactions, we generated VN-tagged Anks1a deletion mutants lacking the ankyrin repeats (dA), the SAM domain (dS) or the PTB domain (dP; Supplementary Fig. 1d). Although all three mutants are expressed at similar levels (Supplementary Fig. 1e), loss of the ankyrin repeats (dA) markedly reduces overall fluorescence complementation (Fig. 1f, second panel) in comparison with the dS and dP mutants (Fig. 1f, third and fourth panels). Together, these data suggest the ankyrin repeats may be required for a direct interaction between Anks1a and EphA2.

Consistent with these BiFC results, glutathione *S*-transferase (GST)-tagged ANK protein effectively precipitates EphA2 from CT26 cell extracts. This is in contrast to the results obtained with GST-SAM and GST-PTB proteins (Supplementary Fig. 1f). When ectopically expressed in 293 cells, GST-ANK also associates with EphA2-GFP but not N-cadherin-GFP (Supplementary Fig. 1g). Consistent with a critical role for the ANK domain in binding EphA2, we observed that co-expression of EphA2-VC with the Numb PTB adaptor protein, which lacks an ANK domain, produces no detectable BiFC signal (Supplementary Fig. 1c, fourth panel). Anks1a seems capable of interacting with various Eph receptors, since Anks1a-VN also produces intense, mainly cytoplasmic BiFC signal with EphB2 (Supplementary Fig. 1c, fifth panel). Using serial deletions of the EphA2 cytoplasmic region in the BiFC assay, we found EphA2's interaction with Anks1a requires its tyrosine kinase domain (TKD) (Supplementary Fig. 1h–j). Together, our results suggest the ANK domain of Anks1a associates with the TKD of EphA2 in the ER.

Next, we used subcellular markers to determine whether the Anks1a/EphA2 complex producing the BiFC fluorescent signal is localized to the ER. Indeed, the Anks1a/EphA2 complex co-localizes with the ER markers, Calnexin, Calregulin and ER tracker, but not with Golgi markers (Fig. 1g,h). This reveals that EphA2 interacts with Anks1a in the ER rather than in the Golgi. Deletion of Anks1a's PTB domain but not its SAM domain increases intracellular staining for the Anks1a/EphA2 complex (Supplementary Fig. 1k). To identify the Anks1a domains required for cell surface localization of EphA2, we transfected MEFs from *Anks1a*-null mutant embryos with various Anks1a deletion mutants. While expression of WT Anks1a restores cell surface binding of ephrinA5-Fc to the level observed in WT MEFs, cells transfected with each of the Anks1a deletion mutants are indistinguishable from untransfected *Anks1a* mutant MEFs (Fig. 1i,j). This suggests that the different Anks1a domains play distinct roles in the transport of EphA2 from the ER to the cell surface.

To further visualize the export of EphA2 from the ER, we transfected WT or *Anks1a*-null MEF cells with EphA2-GFP (Supplementary Fig. 2a–g). Some GFP-positive vesicles in WT MEFs co-localize with the COPII vesicle markers Sec23 and Sec31 (Supplementary Fig. 2e,f), implying that these are ER-derived transport vesicles. In addition, the relative size of these GFP-positive vesicles is twofold larger in *Anks1a*-null MEFs despite the fact that microtubule organization remains unaltered (Supplementary Fig. 2a,b). Using the ER-tracker dye, we observed a sixfold increase in the number of GFP-positive structures situated around the ER in *Anks1a*-null mutant MEFs when compared with WT MEFs (Supplementary Fig. 2c,d). This concentration of EphA2-GFP staining around the ER implies a problem with ER exit. Consistent with this possibility, EphA2-GFP co-localization with Sec23A or Sec31A is reduced by more than fivefold in *Anks1a*-null mutant MEFs (Supplementary Fig. 2e–g). Together, these results suggest EphA2-GFP is not efficiently packaged into COPII vesicles in the absence of Anks1a.

### Anks1a PTB domain binding to Sec23 regulates EphA2 ER export

**Figure 2:**
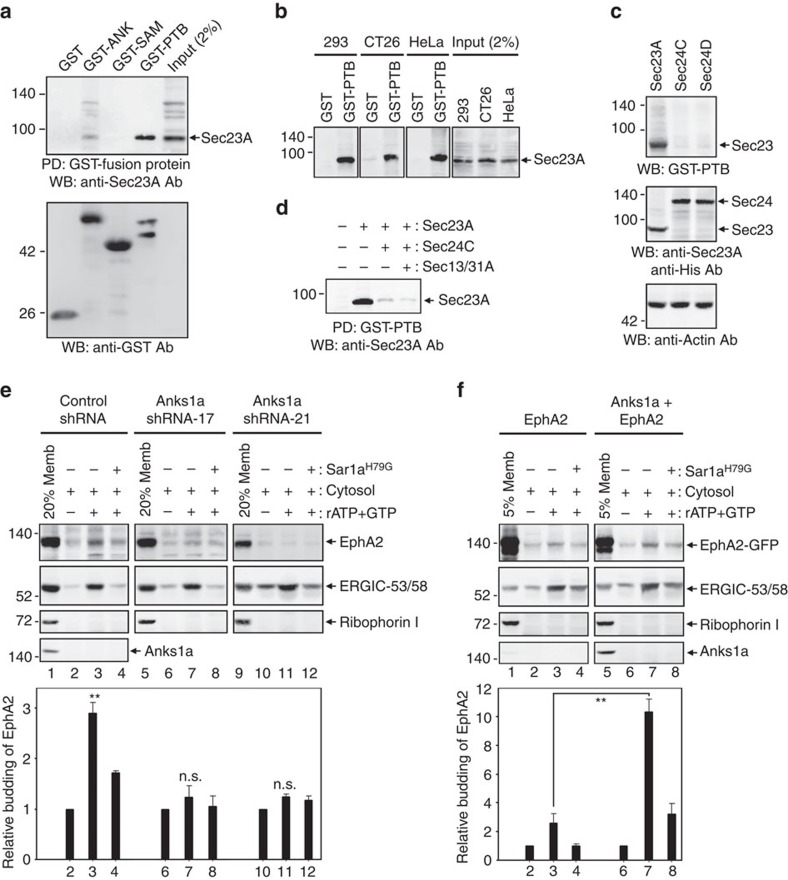
The Anks1a PTB domain binds directly to Sec23 and regulates EphA2 packaging into COPII vesicles. (**a**) CT26 cell lysates were incubated with GST-fusion proteins and the bound proteins were detected with an anti-Sec23A antibody (top) or anti-GST antibody (bottom). (**b**) The indicated cell lysates were mixed with GST-PTB and the bound proteins were detected with an anti-Sec23A antibody. (**c**) Sf9 cells were infected with baculoviruses expressing the indicated proteins. Each Sf9 cell lysate was subjected to SDS– polyacrylamide gel electrophoresis and the separated proteins were probed directly with GST-PTB (top) or the indicated antibodies (middle and bottom). Note that the Sec24 proteins were tagged with a His epitope. (**d**) Sec23 was bound to GST-PTB and then the protein complexes were further incubated with Sf9 cell lysates containing other COPII components on ice for 1 h. Then, GST-PTB protein complexes were washed extensively before detection with an anti-Sec23A antibody. (**e**) Transfected CT26 cells were permeabilized with digitonin for the preparation of ER microsomes. Memb, ER membrane fraction (first lane of each panel); rATP+GTP, ATP regeneration system; Sar1a^H79G^, an inhibitor for COPII-specific vesicle budding. The addition of cytosol (a source for COPII) without an ATP regeneration system was used as a control (second lane of each panel). The budding efficiency of EphA2 in each condition (third and fourth lanes of each panel) was normalized to the level of EphA2 in the control lane. Results were reproducible in five independent experiments (*n*=5). **P<0.01, one-way analysis of variance test. (**f**) Experiments were performed essentially as in **e**, except that the indicated constructs were transfected into HEK293T cells (*n*=4). **P<0.01, Student's *t*-test. n.s., not significant. Data represent means±s.e.

Next, we tested whether the Anks1a PTB domain interacts with COPII transport vesicles. To do so, we used GST fusions to each of the Anks1a domains to pull-down proteins derived from COPII vesicles (Fig. 2a,b). While the PTB domain of Anks1a shows high affinity for Sec23, the ANK and SAM domains do not (Fig. 2a). GST-PTB fusions effectively precipitate Sec23 from three independent cell lines (Fig. 2b). GST-PTB fusions also precipitate other COPII components (that is, Sec24, Sec13 and Sec 31) at levels corresponding to less than the amount of each protein in 2% of total cell lysate (Supplementary Fig. 3a). To determine whether the Anks1a PTB domain binds directly to Sec23, we overexpressed each COPII vesicle component in Sf9 cells using baculoviruses and probed lysates from these cells with GST-PTB in far-western analyses. This allowed us to confirm specific binding of the Anks1a PTB domain to Sec23, but not to any of the other components of COPII vesicles (Fig. 2c). In addition, Sec24 or Sec13/Sec31 complexes effectively compete with Sec23 for binding to the Anks1a PTB (Fig. 2d). Importantly, the co-localization of Anks1a with Sec23 is threefold higher than its co-localization with ER markers (Supplementary Fig. 3b,c). We also found that co-localization of the Anks1a/EphA2 complex with Sec23 is fivefold higher when its co-localization with Rab11 is used as a control (Supplementary Fig. 3d,e). Together, these results strongly suggest Anks1a regulates EphA2 ER export via a direct interaction of its PTB domain with Sec23.

Next, we used the transport vesicle formation assay^23^ to determine whether EphA2 is packaged into COPII vesicles in CT26 cells (Fig. 2e). We used ERGIC-53, a protein cargo that cycles between the ER and Golgi, as a positive control, and Ribophorin I, an ER resident protein, as a negative control^24^,^25^. As expected, cytosol stimulates the packaging of EphA2 and ERGIC-53 but not Ribophorin I into transport vesicles, but only in the presence of an ATP regeneration system (left panels, lane 3). EphA2 and ERGIC-53 packaging is inhibited by a dominant-negative form of Sar1a (lane 4), consistent with its essential role in COPII vesicle formation^26^. Since Anks1a is barely detectable in COPII vesicles, it does not seem to enter mature COPII carriers (left panels, the bottom one). Importantly, Anks1a knockdown in CT26 cells using short hairpin RNAs (shRNAs) significantly reduces the packaging efficiency of EphA2 but not ERGIC-53 (Supplementary Fig. 3f; Fig. 2e).

We next examined the effect of Anks1a overexpression on EphA2 packaging into transport vesicles (Fig. 2f). For this experiment, we performed a budding assay using microsomes from EphA2-GFP vector-transfected 293 cells. 293 cells endogenously express moderate levels of Anks1a (left panels). As expected, co-transfection with Anks1a stimulates the packaging of EphA2-GFP but not ERGIC-53 (right panels).

We further asked whether deletion of the Anks1a ANK or PTB domains affects the export of EphA2 from the ER. Anks1a lacking either its ANK or PTB domains is nonfunctional in packaging EphA2 into COPII vesicles (Supplementary Fig. 3g). In fact, although Anks1a lacking its PTB domain is well detected in ER microsomes, Anks1a lacking its ANK domain is barely detectable, supporting our previous finding that the ANK domain is critical for EphA2 binding (Supplementary Fig. 3h).

Consistent with a role for Anks1a in COPII-mediated anterograde transport from the ER, we observed abnormally distended ER structures in transmission electron micrographs of *Anks1a*-null mutant MEF cells (Supplementary Fig. 4a,b). In addition, the budding vesicles emerging from the ER in *Anks1a* mutant MEFs are much larger than those in WT MEFs (Supplementary Fig. 4a,c). We also observed abnormal Golgi stacks and markedly (fivefold) reduced lysosomes in *Anks1a* mutant MEF cells, suggesting the loss of Anks1a function in the ER also has a general effect on the endomembrane system (Supplementary Fig. 4d–f).

### Phosphorylation of Anks1a is required for EphA2 ER export

**Figure 3:**
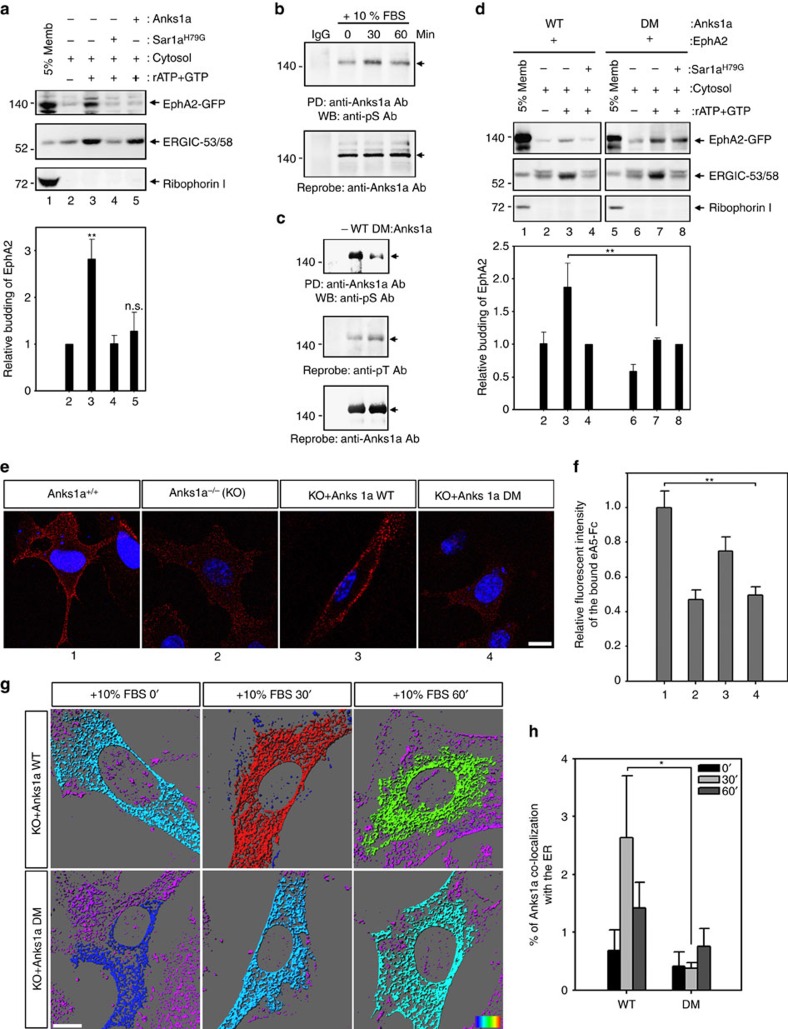
Serine phosphorylation of Anks1a is important for the ER export of EphA2. (**a**) Experiments were performed essentially as in Fig. 2f, except that purified Anks1a protein (His tagged) was added to the budding assay (*n*=4). **P<0.01, one-way analysis of variance (ANOVA) test. (**b**) Serum-starved CT26 cells were stimulated with 10% FBS for the indicated times. Each cell lysate was incubated with an anti-Anks1a antibody and the bound proteins were detected with an anti-phosphoserine (pS) antibody (top panel). (**c**) HEK293T cells were transfected with the indicated constructs and experiments were performed as described in **b**. Note that serine residues 647 and 663 were replaced with alanine in the Anks1a-DM mutant. (**d**) Experiments were performed as described in Fig. 2f except for the substitution of the indicated Anks1a constructs. It is unclear why addition of the ATP regeneration system induces COPII-independent budding of EphA2 in Anks1a-DM-transfected cells (top panel, lanes 7 and 8). This necessitated the use of Sar1a^H79G^ as a control for the normalization of EphA2 budding in other conditions (*n*=4). (**e**,**f**) Experiments were performed essentially as described in Figure 1i. Scale bar, 10 μm. **P<0.01, one-way ANOVA test. (**g**,**h**) *Anks1a* knockout (KO) MEF cells were transfected with the indicated Anks1a constructs. Forty-eight post transfection, cells were starved for 2 h and stimulated with 10% FBS for the indicated times. Then, the cells were immunostained with both anti-Anks1a and anti-Calregulin antibodies. Co-localization in each panel is presented in a pseudo-coloured heat map. Co-localization efficiency of Anks1a with ER was calculated using Imaris (*n*=15 for each case). Scale bar, 10 μm. *P<0.05, Student's *t*-test. n.s., not significant. Data represent means±s.e.

Since overexpression of Anks1a in 293 cells enhances EphA2 loading into COPII vesicles, we tested whether purified Anks1a recapitulates this result in an *in vitro* budding assay. First, we confirmed the Anks1a PTB domain alone competes with endogenous Anks1a for binding to budding ER microsomes (Supplementary Fig. 5a). We then found, however, that full-length Anks1a purified from Sf9 cells effectively blocks the loading of EphA2 into COPII vesicles (Supplementary Fig. 5b; Fig. 3a). We next performed a tryptophan (Trp) fluorescence assay. We loaded liposomes with Sar1-GTP and measured the intrinsic Trp fluorescence of Sar1 after the addition of Sec23/24 in the presence or absence of purified Anks1a. As expected, the addition of Sec23/24 to Sar1-GTP-containing liposomes increases Sar1 GTPase activity (Supplementary Fig. 5c). The addition of equimolar Anks1a, however, abolishes Sec23/24 GAP activity. Although Anks1a purified from Sf9 cells inhibits Sec23's GAP activity, we hypothesized that post-translationally modified Anks1a may stimulate the packaging of EphA2 into COPII vesicles in mammalian cells. Human Anks1a is known to be phosphorylated on serine residues 647 and 663 (ref. 9). We, too, found evidence of serine phosphorylation of Anks1a in CT26 cells on serum stimulation (Fig. 3b). By treating cells with appropriate chemical inhibitors, we found serum-induced Anks1a phosphorylation is mediated by Erk rather than mammalian target of rapamycin (mTOR) (Supplementary Fig. 5d). To further investigate the role Anks1a phosphorylation plays in the packaging of EphA2 into COPII vesicles, we generated an Anks1a-S647A/S663A double mutant (Ser DM). As expected, the phosphoserine signal in 293 cells expressing the Anks1a-Ser DM mutant is significantly lower than that of cells expressing WT Anks1a (Fig. 3c, first panel). In contrast, the signal for phosphothreonine remains unaltered (second panel). Indeed, we next observed that Anks1a-Ser DM is defective in stimulating the packaging of EphA2 into COPII vesicles (Fig. 3d). The level of Anks1a in ER microsomes is significantly reduced in Anks1a-Ser DM-transfected cells (Supplementary Fig. 5e). We also found Anks1a-Ser DM is partially defective in its interaction with EphA2 in co-transfected cells: Anks1a-Ser DM binding to EphA2 is roughly 50% of that of WT Anks1a (Supplementary Fig. 5f,g). More importantly, Anks1a-Ser DM cannot rescue the cell surface localization of EphA receptors in *Anks1a*-null mutant MEFs (Fig. 3e,f). We also observed a significant increase in the co-localization of Anks1a staining with the ER marker Calregulin on serum stimulation of *Anks1a*-null MEFs transfected with WT *Anks1a* but not with the *Anks1a-Ser DM* mutant (Fig. 3g,h). Together, these results strongly suggest serine phosphorylation of Anks1a is important for Anks1a's selective localization to the ER where it regulates COPII vesicle biogenesis.

### EphA2 ER export is critical for tumour growth

Since CT26 cells express undetectable levels of ephrin ligands, CT26 cells are a good experimental model for EphA2-mediated tumour growth. Indeed, CT26 cells expressing *EphA2*-specific shRNAs show reduced Erk activity, reduced colony-forming ability in soft agar and reduced tumour growth on transplantation into syngenic Balb/c mice (Supplementary Fig. 6a–d).

**Figure 4:**
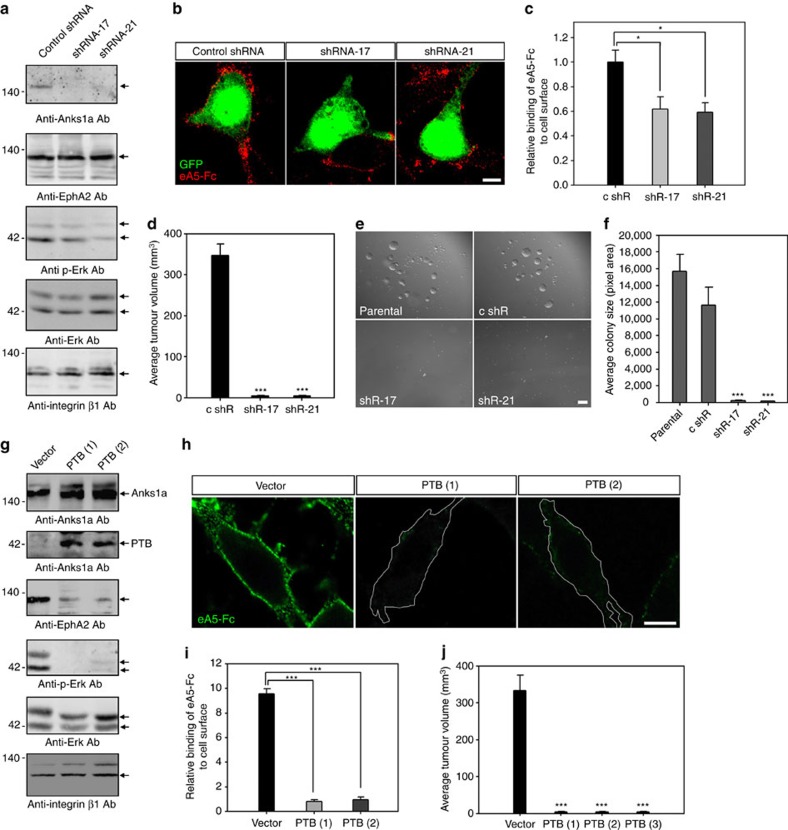
Downregulation of Anks1a reduces the tumour growth of CT26 cells. (**a**) Lysates from CT26 cells infected with each lentivirus were immunoblotted with the indicated antibodies. (**b**,**c**) CT26 cells were subjected to surface-binding assays using ephrinA5-Fc as described in Fig. 1c. Green fluorescence indicates cells infected with lentivirus. Scale bar, 10 μm. *P<0.05, Student's *t*-test. (**d**) Virus-infected CT26 cells were transplanted into Balb/c female mice by subcutaneous injection. After 4 weeks, tumour volumes were calculated. ***P<0.001, Student's *t*-test. (**e**,**f**) Virus-infected CT26 cells were seeded on 0.5% agarose containing medium. After 3 weeks, the size of each colony was measured using ImageJ. Scale bar, 50 μm. ***P<0.001, Student's *t*-test. (**g**–**j**) Experiments were performed essentially as described in **a**–**f**, except for the substitution of CT26 cells stably expressing the PTB domain of Anks1a. Scale bar, 10 μm. ***P<0.001, Student's *t*-test. Data represent means±s.e.

To investigate EphA2-mediated tumour growth, we used CT26 cell lines expressing one of two shRNAs (shRNA-17 or -21) specific to *Anks1a* (Fig. 4a, first panel). Although neither shRNA reduces the total level of EphA2 (second panel), both reduce Erk activity when compared with the expression of control shRNAs (third and fourth panels). Knockdown of Anks1a also reduces cell surface binding of ephrinA5-Fc approximately twofold, indicating a reduction in EphA2 binding (Fig. 4b,c). We also transplanted CT26 cells expressing *Anks1a*-specific shRNAs into syngenic Balb/c mice. Although control CT26 cells effectively induce tumour formation within 2 weeks, CT26 cells depleted for *Anks1a* do not (Fig. 4d). Similarly, knockdown of *Anks1a* also reduces the ability of CT26 cells to form colonies in a soft agar (Fig. 4e,f).

It is unclear why, but CT26 cells ectopically expressing the Anks1a PTB domain show reduced total EphA2 despite showing no change in the level of endogenous Anks1a (Fig. 4g, third panel). Still, ectopic expression of the Anks1a PTB domain in CT26 cells reduces Erk activity (fourth panel) and markedly reduces (≥10-fold) EphA2 cell surface localization (Fig. 4h,i). Ectopic Anks1a PTB domain expression also inhibits the tumour growth potential of CT26 cells as measured by syngenic transplantation experiments (Fig. 4j). Interestingly, ectopic expression of the Anks1a PTB domain has a much larger effect on Erk activity and tumour growth than *Anks1a*-specifc shRNA expression, suggesting that the Anks1a PTB domain is a strong dominant-negative inhibitor of Anks1a in CT26 cells. It is also possible that the reduced Erk signalling of cells expressing *Anks1a* shRNAs has nothing to do with EphA2. To address this issue, we transfected CT26 cells expressing mouse *Anks1a* shRNAs with a human Anks1a cDNA or with the Ansk1a-Ser DM mutant that is partially defective for binding to EphA2. It is important to note that both of these human cDNAs are resistant to mouse *Anks1a*-specific shRNAs. While the WT Anks1a cDNA restores normal levels of Erk activity, the Anks1a-Ser DM mutant does not (Supplementary Fig. 6e,f). Together, our findings demonstrate Anks1a-regulated EphA2 ER export significantly enhances the level of EphA2 at the cell surface and consequently enhances tumour growth.

### Anks1a plays a crucial role in breast tumorigenesis

**Figure 5:**
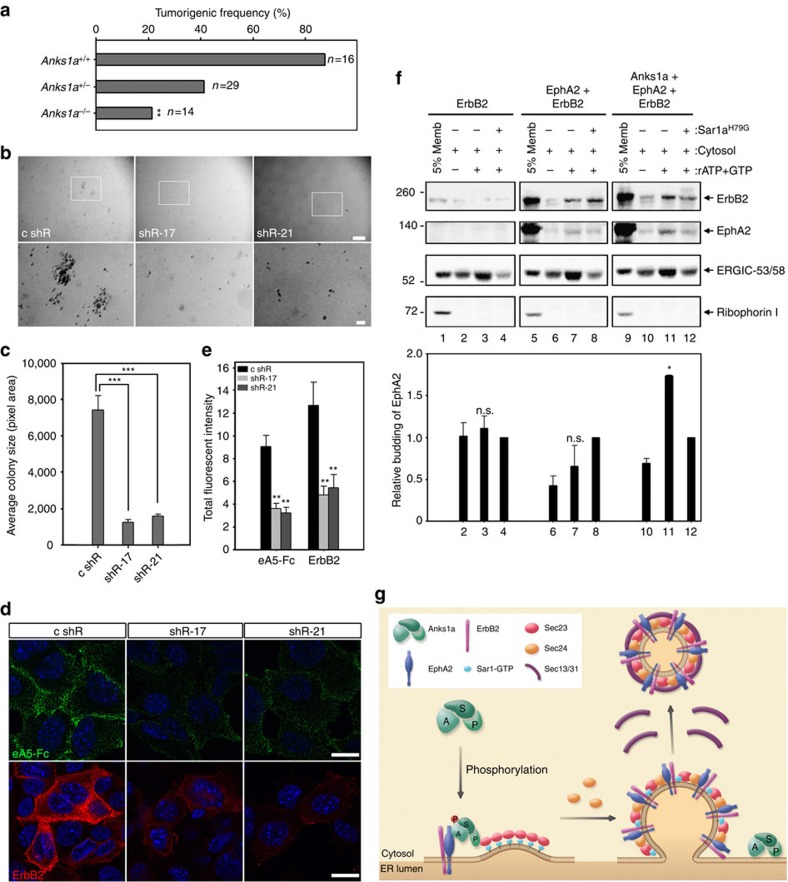
Anks1a influences breast tumorigenesis by regulating the ER export of EphA2–ErbB2 complexes. (**a**) *Anks1a*^*+/−*^ female mice were crossed with *Anks1a*^*+/−*^*; MMTV-Neu* male mice to obtain female mice with the indicated genotypes. All experiments monitoring breast tumour formation were carried out in mice of the FVB genetic background (*n*=16, 29, 14 per genotype). **P<0.01, *χ*^2^-test. (**b**,**c**) PMTCs were isolated from breast tumours of *MMTV-Neu* female mice and transduced with *Anks1a*-specific shRNA lentiviruses. Infected cells were subjected to soft agar colony formation assays as described in Fig. 4e. Scale bar, 250 μm (top panel); 50 μm (bottom panel). ***P<0.001, Student's *t*-test. (**d**,**e**) PMTCs were stained with ephrinA5-Fc or an anti-ErbB2 antibody without permeabilization to detect cell surface receptors as described in Fig. 1c. Scale bar, 20 μm. **P<0.01, Student's *t*-test. (**f**) HEK293 cells were co-transfected with the indicated constructs and COPII vesicle-budding assays were performed as described in Fig. 2f (*n*=4). Note the use of Sar1a^H79G^ as a control for the normalization of the EphA2 budding. *P<0.05, one-way analysis of variance test. (**g**) Model in which Anks1a facilitates the export of EphA2 and ErbB2 RTKs from the ER. In mitogen-activated cells, Ser-647 and Ser-663 of Anks1a are likely the major regulatory phosphorylation sites. Our experiments indicate that serine phosphorylation of Anks1a is critical for its subcellular localization to the ER. Serine phosphorylation may induce a conformational change in Anks1a so it can interact effectively with both EphA2 and Sec23 in the ER. The ANK domain of Anks1a binds to EphA2 or EphA2/ErbB2 complexes, whereas the PTB domain binds to Sec23, a critical component of COPII vesicles. Then, other COPII components (that is, Sec24 and Sec13/31) are recruited to Sec23 in the ERES, possibly competing away Anks1a. This dynamic COPII biogenic process would direct the loading of RTK cargos into growing COPII carrier vesicles. n.s., not significant. Data represent means±s.e.

EphA2 is known to form a complex with ErbB2/HER2/Neu that is important for tumour initiation and metastasis in *MMTV-Neu* transgenic mice^21^. We also observed that BiFC signals indicating ErbB2-VN forms a complex with EphA2-VC in the ER as well as the plasma membrane (Supplementary Fig. 7a). We further examined the effect of the *Anks1a*-null mutation in the context of the *MMTV-Neu* transgenic mouse model for human breast tumour formation^27^. *Anks1a*^*+/−*^ and *Anks1a*^*−/−*^ female *MMTV-Neu* mice have two- and fourfold fewer mammary gland tumours, respectively, than *Anks1a*^*+/+*^ *MMTV-Neu* mice 1 year after birth (Fig. 5a). To determine how directly Anks1a inhibits tumorigenesis in the mammary epithelium, we isolated primary mammary tumour cells (PMTCs) from *MMTV-Neu* females and measured their colony-forming ability on infection with *Anks1a*-specific shRNA lentivirus. Stable expression of two independent *Anks1a*-specific shRNAs markedly reduces Anks1a expression in *MMTV-Neu* PMTCs relative to cells expressing a control shRNA (Supplementary Fig. 7b, first panel). PMTCs with reduced Anks1a expression show no change in the levels of either ErbB2 or EphA2 (second and third panels), but they do show reduced Erk activity (fourth and fifth panels). More importantly, PMTCs with reduced Anks1a expression also form fourfold smaller colonies than PMTCs expressing a control shRNA (Fig. 5b,c). Taken together, our data show that Anks1a is critically involved in the EphA2-mediated ErbB2 signal amplification that plays a role in breast tumorigenesis.

We next examined the cell surface localization of both EphA2 and ErbB2 to identify the mechanism by which Anks1a deficiency inhibits PMTC tumorigenesis. As expected, using an ephrinA5-Fc-binding assay or EphA2-specific antibody staining, we observed 2.5-fold less cell surface localization of EphA2 in cells with reduced Anks1a expression (Fig. 5d, top panels; Fig. 5e; Supplementary Fig. 7c,d). In addition, the same cells also show threefold less cell surface staining of ErbB2 (Fig. 5d, bottom panels; Fig. 5e). Since the binding of Anks1a-Ser DM mutant to EphA2 is less than twofold that of WT Anks1a (Supplementary Fig. 5f,g), we asked whether expression of the Anks1a-Ser DM mutant in *Anks1a*^*−/−*^; *MMTV-Neu* PMTCs affects ErbB2 cell surface localization and signalling. Unlike WT Anks1a, the Anks1a-Ser DM mutant does not restore the cell surface localization of EphA2 or ErbB2 (Supplementary Fig. 7e,f). The Anks1a-Ser DM mutant also cannot restore the colony-forming ability of *Anks1a*^*−/−*^; *MMTV-Neu* PMTCs in soft agar (Supplementary Fig. 7g,h) or Erk signalling (Supplementary Fig. 7i). Together, these results indicate that the specific interaction between Anks1a and EphA2 is important for breast tumour growth in MMTV-Neu mouse model.

We next used the SK-BR-3 human breast cancer cell line with ErbB2 amplification to see whether Anks1a plays a role in EphA2-mediated ErbB2 signalling. Erk activity is significantly reduced in cells stably expressing two different human *Anks1a*-specific shRNAs than in cells expressing control shRNAs (Supplementary Fig. 8a). Knockdown of human *Anks1a* markedly reduces cell surface binding of both ephrinA5-Fc- and ErbB2-specific antibodies, indicating defective cell surface localization of EphA2 and ErbB2 (Supplementary Fig. 8b,c). Knockdown of Anks1a also reduces the ability of SK-BR-3 cells to form colonies in a soft agar (Supplementary Fig. 8d,e).

Finally, we asked whether Anks1a-dependent export of ErbB2 from the ER depends on EphA2. We found that, although expression of EphA2 increases the level of ErbB2 in the ER (Fig. 5f, top panels, lanes 1, 5 and 9), this elevated steady-state level of ErbB2 is independent of the level of Anks1a. We found, however, that the packaging of ErbB2 into COPII vesicles requires both EphA2 and Anks1a (top panels, lane 11). These effects seem specific, as ERGIC-53 packaging appears normal in all transfections. ErbB2 is poorly packaged into COPII vesicles in cells co-transfected with both ErbB2 and EphA2 (second panels, lanes 7 and 8) and in cells co-transfected with ErbB2 and Anks1a (Supplementary Fig. 8f–h). Together, these results suggest that ErbB2 is stabilized by its association with EphA2 in the ER, and that ErbB2's exit from the ER is facilitated by the interaction between EphA2 and Anks1a (Fig. 5g).

## Discussion

In this study, we identified dual functions in the ER for the cytosolic phosphoprotein Anks1a. Not only does Anks1a promote the exit of the EphA2 RTK from the ER, but it also interacts with Sec23 to direct its loading into COPII vesicles (Fig. 5g). ErbB2, an important therapeutic target for breast cancer, is known to interact with EphA2 in promoting tumour progression^21^,^28^. We found that the interaction between ErbB2 and EphA2 in the ER promotes ErbB2 stability, and that the interaction of EphA2 with Anks1a directs the loading of ErbB2 into growing COPII vesicles. Our identification of Anks1a as a novel regulator of the ER export of the EphA2/ErbB2 RTK complex provides important insights into breast tumorigenesis and into the mechanism regulating COPII vesicle biogenesis in response to the cellular secretory demands of RTKs.

According to our *in vitro* budding assay, purified Anks1a inhibits the budding of EphA2. We also found that purified Anks1a inhibits Sec23's GAP activity towards the Sar1 GTPase. These data contradict the stimulation of EphA2 loading into COPII vesicles by Anks1a in the COPII vesicle budding assay. This conflict may be explained by post-translational modification of Anks1a. Unmodified Anks1a, the predominant form in the cytosol, binds tightly to Sec23 and inhibits Sec23/24 complex formation. A post-translationally modified form of Anks1a may instead be localized to the ER, regulating COPII-mediated EphA2 ER export. Consistent with this prediction, we demonstrated the importance of Anks1a serine phosphorylation for its localization to the ER and for its role in the packaging of EphA2 into COPII carriers. 14-3-3 proteins interact with several phosphorylated binding motifs of Anks1a, including serines 647 and 663 (ref. 9). It remains unclear, however, whether binding of 14-3-3 proteins to Anks1a is physiologically relevant to Anks1a's role in the export of RTK cargos from the ER.

Since serum stimulation induces serine phosphorylation of Anks1a, the internalization of RTKs occurring on cognate ligand binding may trigger Anks1a to upregulate RTK export from the ER. Interestingly, serum-induced phosphorylation of Anks1a is blocked by an Erk kinase inhibitor. This suggests RTK-dependent growth factor signalling may stimulate anterograde trafficking from the ER to the plasma membrane. Once Anks1a moves from the cytoplasm to the ER in response to serine phosphorylation, it may be further stabilized in the ER via a direct association of its ANK domain with EphA2 or via its indirect interaction with ErbB2. These interactions would facilitate the selective concentration of these RTK cargos into ERES, a preliminary step in the cargo loading of budding COPII vesicles. Indeed, Anks1a lacking its ANK domain is neither localized to the ER nor capable of stimulating EphA2's ER export. It is possible serine phosphorylation of Anks1a regulates its own ANK domain. Perhaps, for example, serine phosphorylation of Anks1a allows its ANK domain easier access to EphA2 in the ER. A detailed structure of Anks1a will provide important insight into this mechanism in the future.

As RTK cargos are concentrated in the ERES, the Anks1a PTB domain takes a dynamic role in physically linking cargo to growing vesicles via its binding to Sec23 (ref. 29). We found that the binding of the Anks1a PTB domain to Sec23 can be competitively inhibited by other COPII components. It is thus possible that Anks1a in the ER is engaged in a sort of tug of war with other COPII components for Sec23: as Sec23 is pulled more strongly by Sec24 and subsequently by the Sec13/Sec31 complex, its interaction with Anks1a is gradually lost. It will be interesting to determine whether RTK cargos enter budding COPII vesicles via interactions with Sec24 or other cargo adaptors. Since we found no evidence of Anks1a in mature COPII vesicles, it is likely released from the ER or recycled to influence the packaging of other newly synthesized RTKs. Although some PTB domains of Dab-like PTB proteins are known to bind unphosphorylated NPXY motifs^7^, Sec23 protein lacks any such motif. The identification of this binding site on the Anks1a PTB domain for Sec23 should be addressed by future experiments.

Although we have not determined a detailed mechanism, we have implicated the Anks1a SAM domain in the cell surface localization of RTK cargos. Since the Anks1a SAM domain is known to recognize ubiquitinated proteins and since ubiquitin is an important regulator of COPII coat size, the Anks1a SAM domain may interact with ubiquitinated Sec31 to regulate COPII carrier size^5^,^30^.

*Anks1a*-null mutant MEF cells show partial disruption of the endomembrane system, including the Golgi and lysosomes. This is probably due to inefficient anterograde transport from the ER to the Golgi. In this respect, Anks1a may be involved in transporting other categories of cell surface receptors from the ER. Ankyrin repeats typically fold together to form the solenoid-like ankyrin repeat domain, which is one of the most common protein–protein interaction domains^31^,^32^. Although ankyrin repeat domains are found in many diverse protein families, there is no specific structure known to recognize them. Here we report the ANK region, which contains six ankyrin repeats, is critical for the selective binding of Anks1a to the TKD of EphA2. In the future, we will determine whether this interaction masks the ER retention signal of the EphA2 TKD. Although we have observed binding of Anks1a with various other receptors in co-transfected cells (Supplementary Fig. 8f,g), this binding does not promote their ER export in *in vitro* budding assays (Supplementary Fig. 8h). Likewise, we found that the loss of Anks1a does not affect the cycling of the integral membrane protein ERGIC-53 between the ER and Golgi. We propose, therefore, that Anks1a is a specific, not universal regulator of vesicle cargos. It remains unclear whether other cell surface receptors that are co-immunoprecipitated with Anks1a require EphA2 or other distinct RTKs for their efficient ER export. We propose that each RTK directly bound to Anks1a forms distinct heteromeric complexes with other cell surface receptors in the ER. If true, Anks1a would likely affect their ER export as well. This hypothesis requires further investigation. Reportedly, Anks1a depletion reduces EGF-induced EGFR internalization into recycling endosomes and increases routing to lysosomes^10^. Although Anks1a was barely localized to endosomes or lysosomes in our study, we do not rule out the possibility that Anks1a could act both at the level of the ER and the level of endosomes. A definitive assessment of the relative contribution of these two pools remains to be determined as a future goal.

The efficient and selective export of mature, correctly folded proteins from the ER is critical for organismal health. Here we demonstrated a role for Anks1a in breast tumorigenesis. Specifically, Anks1a facilitates the efficient ER export of EphA2/ErbB2 complexes. *Anks1a* is frequently overexpressed in various cancer cell types, and a recent a genome-wide association study named *Anks1a* among the top 17 genes with single-nucleotide polymorphisms associated with advanced-stage non-small cell lung cancer^33^. It will be interesting to determine whether any of these single-nucleotide polymorphisms are gain-of-function mutations in Anks1a that abnormally promote RTK export from the ER. Since we report the Anks1a ANK domain interacts with the TKD of EphA2, it may represent a novel therapeutic target for the treatment of cancer. In addition, two recent papers have identified *de novo* mutations in *Anks1a* associated with schizophrenia and autism spectrum disorder^34^,^35^. Thus, our detailed molecular examination of the role the Anks1a adaptor plays in the ER export of RTKs may soon provide important insights into the molecular pathogenesis of several human diseases.

## Methods

### Reagents

Recombinant human ephrinA5-Fc was from R&D Systems. Antibodies used in the study were: rabbit anti-Anks1a (BETHYL, KIAA0229, 1:50), Sec16 (BETHYL, KIAA0310, 1:100), phosphoserine (Abcam, ab9332, 1:1,000), Erk (Cell Signaling Technology, 9102, 1:2,000), ERGIC-53 (Sigma Aldrich, E1031, 1:200), actin (Sigma Aldrich, A2066, 1:5,000), HA (Invitrogen, 71-5500, 1:2,000), EphA2 (Santa Cruz Biotechnology, C-20, 1:1,000), N-terminus-specific GFP (MBL, 598, 1:100) and C-terminus-specific GFP (Lifespan Biosciences, LS-C51736, 100), goat anti-Calregulin (Abcam, ab4109, 1:100), Sec23A (Sigma Aldrich, S7696, 1:100), Ribophorin I (Santa Cruz Biotechnology, C-15, 1:1,000), Rab11 (Santa Cruz Biotechnology, C-19, 1:100), mouse anti-GM130 (BD Biosciences, 610822, 1:200), p230 (BD Biosciences, 611280, 1:100), phospho-Erk (Cell Signaling Technology, 9101, 1:2,000), His (Sigma Aldrich, H1029, 1:2,000), EphA2 (Invitrogen, 37-4400, 1:100), ErbB2 (Neomarkers, MA5-12998, 1:100), rat anti-LAMP1 (Santa Cruz Biotechnology, 1D4B, 1:50), chicken anti-Calnexin (Abcam, ab140818, 1:200), and ER-tracker (ThermoFisher Scientific, E12353, 1:500).

Anks1a (BC050847), EphA2 (BC037166.2) and ErbB2 (BC167147.2) cDNAs were purchased from Addgene. Venus C-terminal (pRS303:VC) and N-terminal (pRS306:VN) constructs (Addgene) were used as templates for PCR amplification. The resulting VC or VN PCR products were inserted into the C terminus of Anks1a, EphA2 or ErbB2. Sar1A^H79G^, Sec23A, Sec24 (A/B/C/D), Sec13 and Sec31 were obtained from Dr Randy Schekman's lab (UC Berkeley). *Anks1a-* or *EphA2*-specific shRNA-expressing plasmids were purchased from Sigma Aldrich.

Rapamycin, torin and PD98059 were purchased from Sigma Aldrich.

### Cell culture

HEK293T, CT26 and SK-BR-3 cells were obtained from ATCC and cultured as described^6^. Sf9 cells were purchased from Invitrogen and cultured according to the manufacturer's protocol. PMTCs were obtained from breast tumours of MMTV-Neu animals. Tumours were isolated, washed three times with PBS and minced with a razor. The small pieces of tumour were then moved into 50 ml falcon tubes and incubated with a collagenase solution (0.2% trypsin, 0.2% collagenase A, 5 μg ml^*−*1^ gentamycin and 5% fetal bovine serum in DMEM/F12 medium) for 2 h at 37 °C. Dissociated tumours were collected by centrifugation at 1,500 r.p.m. for 10 min and the supernatant was discarded. The pellet was resuspended with 4 ml of DMEM/F12 containing DNase (2 U ml^*−*1^) for 5 min with shaking at room temperature, and then PMTCs were cultured as described^21^.

For the soft agar formation assay, cultured CT26 cells or PMTCs were incubated with lentivirus-containing medium for 3 days. Before the assay, 1.5% agarose (in DMEM medium containing 10% FBS) was added to 24-well plates. After the base agarose had completely set, infected cells were detached from the dish with trypsin/EDTA and washed with serum-free DMEM. Dissociated cells were resuspended with 2 × DMEM containing 20% FBS and mixed with 1% agarose. Then, cells were seeded onto the base agar. After 3 weeks, images of each well were captured by digital camera (MP5.0-RTV-CLR-10, QImaging). Colony sizes were measured using ImageJ.

### Immunofluorescence and transmission electron microscopy

MEF cells were grown on coverslips and transiently transfected with plasmids using ViaFect (Promega) according to the manufacturer's protocol. After 48 h, the cells were processed for immunofluorescence staining. All subsequent steps were performed at room temperature and all washing steps were carried out with three changes of PBS. Cells on coverslips were fixed with 4% paraformaldehyde and 2% sucrose in PBS for 30 min and washed. Next, the fixed cells were blocked and permeabilized with blocking buffer (0.1% Triton X-100, 5% horse serum and 3% bovine serum albumin in PBS) for 30 min and washed. Then, cells were incubated with the indicated primary antibodies diluted in 3% bovine serum albumin overnight at 4 °C and washed. Next, they were incubated with blocking buffer for 30 min and stained with TRITC or rhodamine-conjugated anti-rabbit or anti-mouse IgG. After 2 h, cells on coverslips were washed and mounted on glass slides in Vectashield mounting medium with 4,6-diamidino-2-phenylindole. Cells were imaged using a Zeiss confocal microscope. Images were analysed using Imaris (Bitplane).

For ultrastructural examination, MEF cells were fixed in 0.1 mol l^*−*1^ sodium phosphate buffer (pH 7.4) containing 2.5% glutaraldehyde at 4 °C. After washing, the cells were post-fixed for 2 h in the same buffer containing 1% OsO_4_. The cells were subsequently washed and dehydrated using graded alcohol and embedded in Epon-Araldite. Thin sections were prepared on an ultra-microtome (MT-X; RMC, Tucson, AZ, USA). The sectioned cells were mounted on copper grids and double-stained with uranyl acetate and lead citrate. Finally, the grids were examined in a H-7600 transmission electron microscope (Hitachi, Japan).

### Protein purification and Trp fluorescence assay

GST fusion protein purification and GST pull-down assays were performed as previously described^5^. In brief, CT26 cells were cultured to 90–100% confluence in 100 mm plates, washed and lysed with PLC lysis buffer. Next, cell lysates were incubated with the indicated GST fusion proteins for 1 h. Then, the cell lysates were bound to glutathione-Sepharose beads for 30 min at 4 °C. After elution, the bound material was separated using SDS– polyacrylamide gel electrophoresis, transferred to polyvinylidene difluoride membranes and subjected to immunoblotting. To generate His-tagged Anks1a, the coding region of human Anks1a was cloned into the pFastBacHTb vector. His-tagged Anks1a was purified using immobilized Ni-NTA affinity chromatography from lysates of baculovirus-infected insect cells (2 l culture; Bac-to-Bac expression system, Invitrogen).

For the real-time Trp fluorescence assay, liposomes were prepared with an optimal major-minor lipid composition for COPII recruitment as described^36^. In brief, a phospholipid mixture (in chloroform) was evaporated in a glass tube using rotary evaporation at 37 °C. As it formed, the lipid film was hydrated in 1 ml HKM buffer (20 mM HEPES (pH 7.2), 110 mM KOAc and 2 mM MgOAc) at room temperature for 2 h. Liposomes with 10% cholesterol were incubated with 1.5 μM Sar1 and 30 μM GTP. After 1 h, Sec23A/24C with or without purified Anks1a was added. Then, the conversion of Sar1-GTP to Sar1-GDP was monitored using a spectrofluorometer (FP-750, JASCO).

### *In vitro* COPII vesicle formation assay

CT26 cells were cultured in 100 mm plates to 90–100% confluence for the detection of endogenous EphA2 expression. HEK293T cells grown in 100 mm plates were transiently transfected with the indicated plasmids. After 48 h, the cells were washed in PBS, removed from the plates with trypsin and washed in B88-0 buffer (20 mM HEPES (pH 7.2), 250 mM sorbitol, 150 mM KOAc and 5 mM MgOAc) containing 10 μg ml^*−*1^ soybean trypsin inhibitor. The vesicle budding assay was performed as previously described, with some modifications^23^. In brief, the cells were permeabilized with 40 μg ml^*−*1^ digitonin for 5 min in ice-cold B88-0, washed and resuspended in 100 μl B88-0. To form vesicles, donor membranes were mixed with 4 mg ml^*−*1^ mouse liver cytosol, an ATP regeneration system (40 mM creatine phosphate, 0.2 mg ml^*−*1^ creatine phosphokinase and 1 mM ATP), and 0.2 mM GTP followed by a 1 h incubation at 30 °C. Purified Sar1A^H79G^ and GST-PTB proteins (1 μg of each) were added to the budding reactions for inhibition and competition, respectively. Newly formed vesicles were separated from the donor membranes by centrifugation at 15,000 r.p.m. for 15 min. Vesicle-containing supernatants were transferred to 200 μl PCR tubes (subsequently inserted into 13.2 ml Ultra-Clear Centrifuge tubes (Beckman)) and vesicles were collected by centrifugation at 32,000 r.p.m. in a SW41 rotor (LE-80 K, Beckman) for 114 min. Vesicle pellets were resuspended in 35 μl of solubilization buffer. Original donor membranes and vesicle fractions were resolved via SDS– polyacrylamide gel electrophoresis, transferred onto polyvinylidene difluoride membranes and subjected to immunoblotting. All uncropped western blots can be found in Supplementary Fig. 9.

### Mice and *in vivo* tumour studies

*Anks1a*^*−/−*^ mice were described previously^5^. *Anks1a*^*−/−*^ mice in the C57BL/6 background were backcrossed with friend leukemia virus B (FVB) animals for five to seven generations before crossing with *MMTV-Neu* mice (The Jackson Laboratory) in the inbred FVB background. Anks1a genotyping was performed using PCR analysis of tail genomic DNA with the following primers: 5′-TGAAGGCACATGACCCTGAG-3′ (forward1), 5′-ATGTCATAGCTGTTTCCTGT-3′ (forward2), and 5′-ACAGCGTTTGCATCTTGCTG-3′ (reverse). The *Neu* transgenes were detected by PCR using primers and conditions recommended by Jackson Laboratories. Age-matched littermates were monitored for breast tumour formation. All mice use and studies were approved by the Sookmyung Women's University Animal Care and Use Committee.

### Data availability

We declare that the data supporting the findings of this study are available within the article and its Supplementary Information files and from the authors on request.

## Additional information

**How to cite this article:** Lee, H. *et al*. Anks1a regulates COPII-mediated anterograde transport of receptor tyrosine kinases critical for tumorigenesis. *Nat. Commun.* 7:12799 doi: 10.1038/ncomms12799 (2016).

## Supplementary Material

Supplementary Information Supplementary Figures 1-9

## References

[b1] Casaletto J. B. & McClatchey A. I. Spatial regulation of receptor tyrosine kinases in development and cancer. Nat. Rev. Cancer 12, 387–400 (2012). 22622641 10.1038/nrc3277 PMC3767127

[b2] Lemmon M. A. & Schlessinger J. Cell signaling by receptor tyrosine kinases. Cell 141, 1117–1134 (2010). 20602996 10.1016/j.cell.2010.06.011 PMC2914105

[b3] Geva Y. & Schuldiner M. The back and forth of cargo exit from the endoplasmic reticulum. Curr. Biol. 24, R130–R136 (2014). 24502791 10.1016/j.cub.2013.12.008

[b4] Ghersi E., Noviello C. & D'Adamio L. Amyloid-beta protein precursor (AbetaPP) intracellular domain-associated protein-1 proteins bind to AbetaPP and modulate its processing in an isoform-specific manner. J. Biol. Chem. 279, 49105–49112 (2004). 15347684 10.1074/jbc.M405329200

[b5] Kim J. . The SAM domains of Anks family proteins are critically involved in modulating the degradation of EphA receptors. Mol. Cell. Biol. 30, 1582–1592 (2010). 20100865 10.1128/MCB.01605-09 PMC2838079

[b6] Shin J., Gu C., Park E. & Park S. Identification of phosphotyrosine binding domain-containing proteins as novel downstream targets of the EphA8 signaling function. Mol. Cell. Biol. 27, 8113–8126 (2007). 17875921 10.1128/MCB.00794-07 PMC2169194

[b7] Uhlik M. T. . Structural and evolutionary division of phosphotyrosine binding (PTB) domains. J. Mol. Biol. 345, 1–20 (2005). 15567406 10.1016/j.jmb.2004.10.038

[b8] Pandey A. . Cloning of a novel phosphotyrosine binding domain containing molecule, Odin, involved in signaling by receptor tyrosine kinases. Oncogene 21, 8029–8036 (2002). 12439753 10.1038/sj.onc.1205988

[b9] Zhong J. . The interactome of a PTB domain-containing adapter protein, Odin, revealed by SILAC. J. Proteomics 74, 294–303 (2011). 21081186 10.1016/j.jprot.2010.11.006 PMC3205450

[b10] Tong J. . Odin (ANKS1A) modulates EGF receptor recycling and stability. PLoS ONE 8, e64817 (2013). 23825523 10.1371/journal.pone.0064817 PMC3692516

[b11] Caster A. H. & Kahn R. A. Recruitment of the Mint3 adaptor is necessary for export of the amyloid precursor protein (APP) from the Golgi complex. J. Biol. Chem. 288, 28567–28580 (2013). 23965993 10.1074/jbc.M113.481101 PMC3789957

[b12] Rogelj B., Mitchell J. C., Miller C. C. & McLoughlin D. M. The X11/Mint family of adaptor proteins. Brain Res. Rev. 52, 305–315 (2006). 16764936 10.1016/j.brainresrev.2006.04.005

[b13] Shrivastava-Ranjan P. . Mint3/X11gamma is an ADP-ribosylation factor-dependent adaptor that regulates the traffic of the Alzheimer's Precursor protein from the trans-Golgi network. Mol. Biol. Cell 19, 51–64 (2008). 17959829 10.1091/mbc.E07-05-0465 PMC2174186

[b14] Hill K. . Munc18 interacting proteins: ADP-ribosylation factor-dependent coat proteins that regulate the traffic of beta-Alzheimer's precursor protein. J. Biol. Chem. 278, 36032–36040 (2003). 12842896 10.1074/jbc.M301632200

[b15] Brantley-Sieders D., Schmidt S., Parker M. & Chen J. Eph receptor tyrosine kinases in tumor and tumor microenvironment. Curr. Pharm. Des. 10, 3431–3442 (2004). 15544526 10.2174/1381612043383160

[b16] Chen J., Song W. & Amato K. Eph receptor tyrosine kinases in cancer stem cells. Cytokine Growth Factor Rev. 26, 1–6 (2015). 24933439 10.1016/j.cytogfr.2014.05.001 PMC4234705

[b17] Pasquale E. B. Eph receptors and ephrins in cancer: bidirectional signalling and beyond. Nat. Rev. Cancer 10, 165–180 (2010). 20179713 10.1038/nrc2806 PMC2921274

[b18] Zelinski D. P., Zantek N. D., Stewart J. C., Irizarry A. R. & Kinch M. S. EphA2 overexpression causes tumorigenesis of mammary epithelial cells. Cancer Res. 61, 2301–2306 (2001). 11280802

[b19] Pratt R. L. & Kinch M. S. Activation of the EphA2 tyrosine kinase stimulates the MAP/ERK kinase signaling cascade. Oncogene 21, 7690–7699 (2002). 12400011 10.1038/sj.onc.1205758

[b20] Macrae M. . A conditional feedback loop regulates Ras activity through EphA2. Cancer Cell 8, 111–118 (2005). 16098464 10.1016/j.ccr.2005.07.005

[b21] Brantley-Sieders D. M. . The receptor tyrosine kinase EphA2 promotes mammary adenocarcinoma tumorigenesis and metastatic progression in mice by amplifying ErbB2 signaling. J. Clin. Invest. 118, 64–78 (2008). 18079969 10.1172/JCI33154 PMC2129239

[b22] Kerppola T. K. Visualization of molecular interactions by fluorescence complementation. Nat. Rev. Mol. Cell Biol. 7, 449–456 (2006). 16625152 10.1038/nrm1929 PMC2512262

[b23] Shimoni Y. & Schekman R. Vesicle budding from endoplasmic reticulum. Methods Enzymol. 351, 258–278 (2002). 12073349 10.1016/s0076-6879(02)51852-8

[b24] Kim J., Hamamoto S., Ravazzola M., Orci L. & Schekman R. Uncoupled packaging of amyloid precursor protein and presenilin 1 into coat protein complex II vesicles. J. Biol. Chem. 280, 7758–7768 (2005). 15623526 10.1074/jbc.M411091200

[b25] Kim J. . Biogenesis of gamma-secretase early in the secretory pathway. J. Cell Biol. 179, 951–963 (2007). 18056412 10.1083/jcb.200709012 PMC2099203

[b26] Stephens D. J. & Pepperkok R. Differential effects of a GTP-restricted mutant of Sar1p on segregation of cargo during export from the endoplasmic reticulum. J. Cell. Sci. 117, 3635–3644 (2004). 15252131 10.1242/jcs.01269

[b27] Guy C. T. . Expression of the neu protooncogene in the mammary epithelium of transgenic mice induces metastatic disease. Proc. Natl Acad. Sci. USA 89, 10578–10582 (1992). 1359541 10.1073/pnas.89.22.10578 PMC50384

[b28] Rimawi M. F., Schiff R. & Osborne C. K. Targeting HER2 for the treatment of breast cancer. Annu. Rev. Med. 66, 111–128 (2015). 25587647 10.1146/annurev-med-042513-015127

[b29] Fromme J. C., Orci L. & Schekman R. Coordination of COPII vesicle trafficking by Sec23. Trends Cell Biol. 18, 330–336 (2008). 18534853 10.1016/j.tcb.2008.04.006

[b30] Jin L. . Ubiquitin-dependent regulation of COPII coat size and function. Nature 482, 495–500 (2012). 22358839 10.1038/nature10822 PMC3292188

[b31] Li J., Mahajan A. & Tsai M. D. Ankyrin repeat: a unique motif mediating protein-protein interactions. Biochemistry 45, 15168–15178 (2006). 17176038 10.1021/bi062188q

[b32] Mosavi L. K., Cammett T. J., Desrosiers D. C. & Peng Z. Y. The ankyrin repeat as molecular architecture for protein recognition. Protein Sci. 13, 1435–1448 (2004). 15152081 10.1110/ps.03554604 PMC2279977

[b33] Lee Y. . Prognostic implications of genetic variants in advanced non-small cell lung cancer: a genome-wide association study. Carcinogenesis 34, 307–313 (2013). 23144319 10.1093/carcin/bgs356

[b34] De Rubeis S. . Synaptic, transcriptional and chromatin genes disrupted in autism. Nature 515, 209–215 (2014). 25363760 10.1038/nature13772 PMC4402723

[b35] Fromer M. . *De novo* mutations in schizophrenia implicate synaptic networks. Nature 506, 179–184 (2014). 24463507 10.1038/nature12929 PMC4237002

[b36] Futai E. & Schekman R. Purification and functional properties of yeast Sec12 GEF. Methods Enzymol. 404, 74–82 (2005). 16413259 10.1016/S0076-6879(05)04008-5

