# Supplementary material for: Anks1a regulates COPII-mediated anterograde transport of receptor tyrosine kinases critical for tumorigenesis
Source: Nat Commun. 2016 Sep 13;7:12799. doi: 10.1038/ncomms12799 (PMC5027278; doi:10.1038/ncomms12799)
Supplement: Supplementary Information — Supplementary Figures 1-9 [file ncomms12799-s1.pdf]

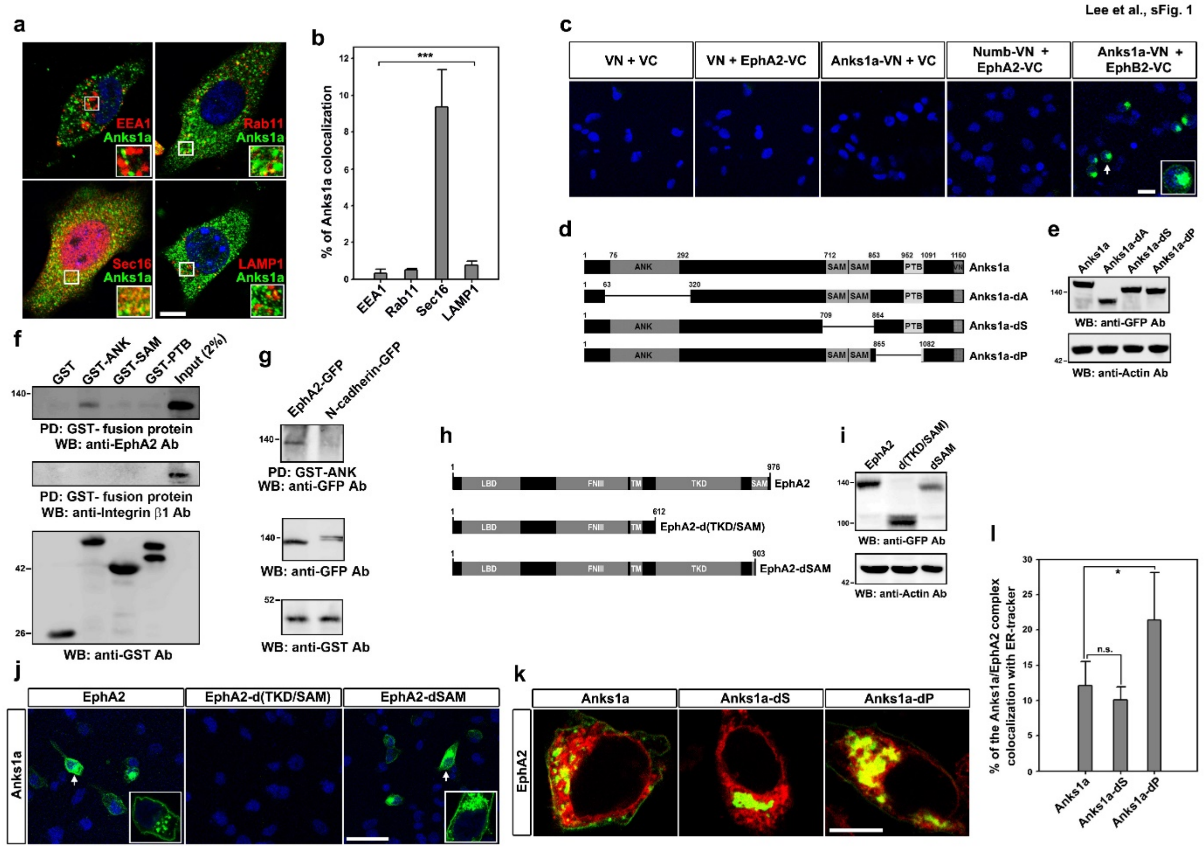

Supplementary Figure 1. Analysis of protein-protein interactions with the BiFC assay. (a and b) Experiments were performed as described in Fig. 1a, except for the substitution of the indicated antibodies. Scale bar, 10 μm. *** *P* < 0.001, one-way ANOVA test. (c) Experiments were performed as described in Fig. 1f, except for the substitution of the indicated constructs. Scale bar, 25 μm. (d) Schematics for the mouse WT Anks1a and various deletion alleles. The C-terminus of each construct was tagged with VN. (e) HEK293 cells were transfected with the indicated constructs and immunoblotted with an anti-GFP antibody. (f) CT26 cell lysates were incubated with GST-fusion proteins. The bound proteins were detected with the indicated antibodies. (g) HEK293 cells were transfected with the indicated GFP-tagged EphA2 or N-cadherin. Proteins bound to GST-ANK were detected with an anti-GFP antibody. (h) Schematics for the human WT EphA2 and various deletion alleles. Note that these constructs were tagged with VC at their C-termini. LBD, ligand binding domain; FNIII, fibronectin type III repeat domain; TM, transmembrane domain; TKD, tyrosine kinase domain; SAM, sterile alpha motif. (i) Experiments were performed as described in e, except for the substitution of the indicated constructs. (j) Experiments were performed as described in Fig. 1f, except for the substitution of the indicated constructs. Scale bar, 50 μm. (k) HEK293 cells were co-transfected with the indicated constructs. Co-transfected cells were incubated with ER-tracker for 1 hour at $37^{o}C$. Scale bar, 10 μm. (l) Co-localization efficiency for the Anks1a/EphA2 complex with ER-tracker was calculated as described in 1h. * *P* < 0.05, student's *t*-test.

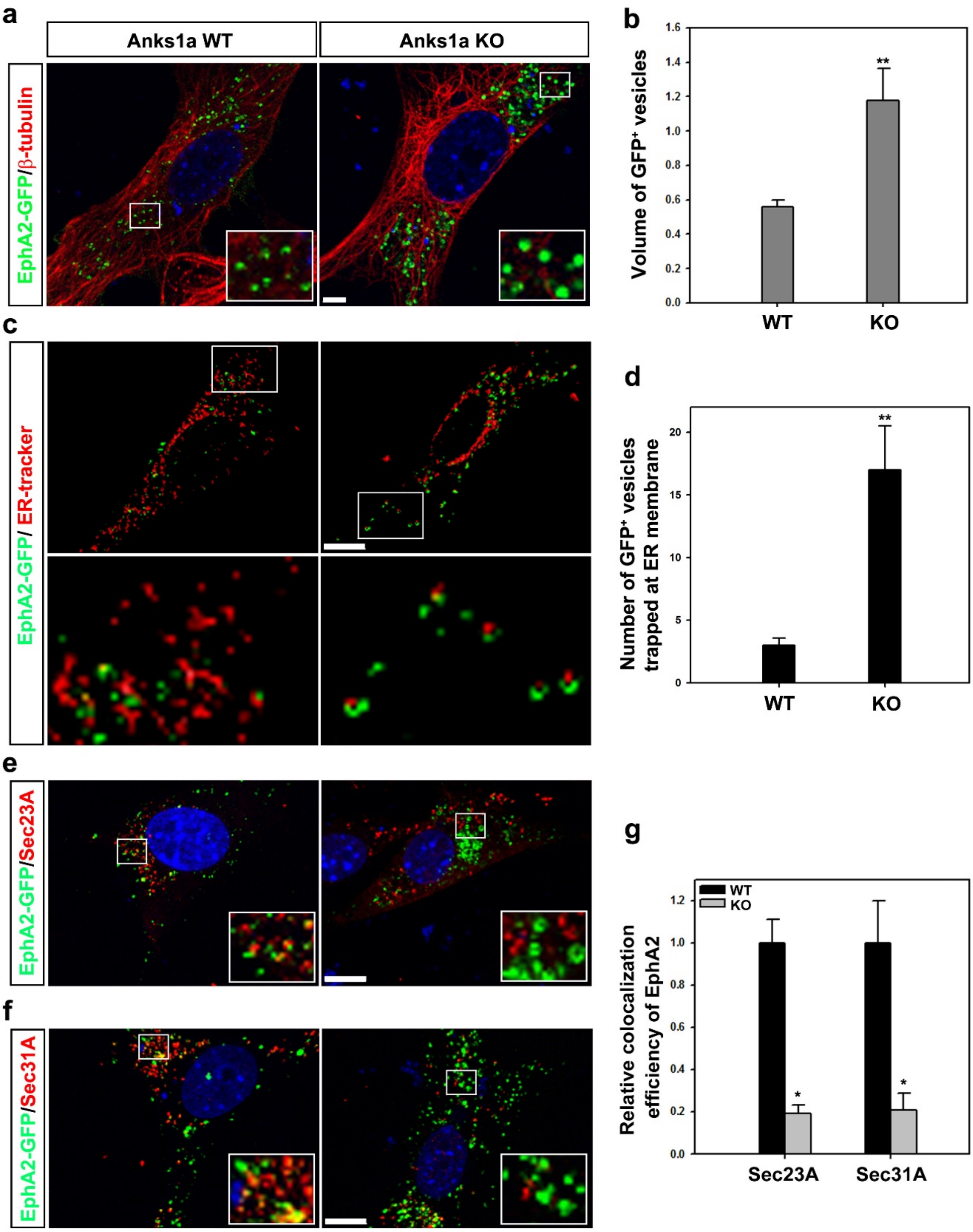

Supplementary Figure 2. Anks1a-deficient MEFs show defective EphA2 ER exit. WT and Anks1a KO MEF cells were transfected with an EphA2-GFP construct. The cells were fixed and stained 48 h after transfection with the indicated markers. (a and b) The volume of GFP-positive vesicles was measured using Imaris (n=15 for each transfection). Scale bar, 10 μm. ** P < 0.01, student's t-test. (c and d) The GFP-positive structures (green) surrounding the ER tracker-stained membrane (red) (marked by arrows in bottom panels) were counted

so the average number per cell could be presented (n=15 for each transfection). Scale bar, 10 μm. ** P < 0.01, student's t-test. (e-g) MEF cells were immunostained with an anti-Sec23A or anti-Sec31A antibody. Co-localization efficiency was calculated as described in Fig. 1b (n=25 for each transfection). Scale bar, 10 μm. * P < 0.05, student's t-test.

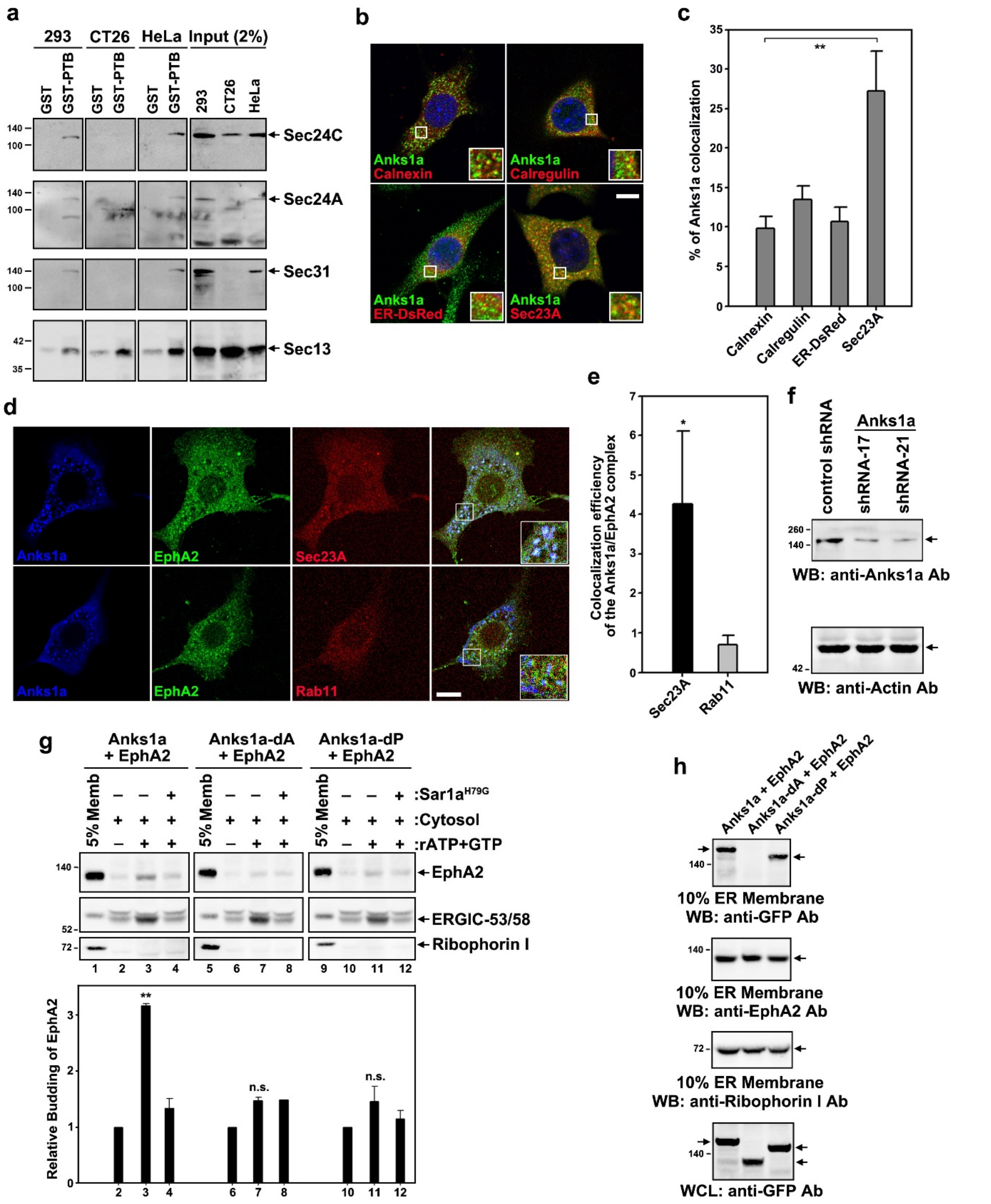

Supplementary Figure 3. Functional analysis of the Anks1a ANK and PTB domains in the ER. (a) Experiments were performed as described in Fig. 2b, except for the substitution of the indicated antibodies. Note that each precipitated protein represented less than 2% of each total cell lysate. (b and c) Experiments were performed as described in Fig. 1a, except for the substitution of the indicated antibodies. Scale bar, 10 μm. ** *P* < 0.01, one-way ANOVA test. (d and e) Experiments were essentially performed as described in Fig. 1a,

except that three different antibodies were incubated at the same time and detected with three different fluorophores (488 nm for Anks1a, 594 nm for EphA2 and 750 nm for Sec23 or Rab11). Scale bar, 10 μm. * $P < 0.05$, student's *t*-test. (f) Lysates from CT26 cells infected with each lentivirus were immunoblotted with the indicated antibodies. (g) Experiments were performed essentially as described in Fig. 2f. ** $P < 0.01$, one-way ANOVA test. (h) ER membrane fractions were isolated from the transfected cells and proteins were detected using the indicated antibodies (from first to third panel). Whole cell lysates (WCL) were subjected to western blot analysis using an anti-GFP antibody. Note that Anks1a tagged with VN was detected with an anti-GFP antibody.

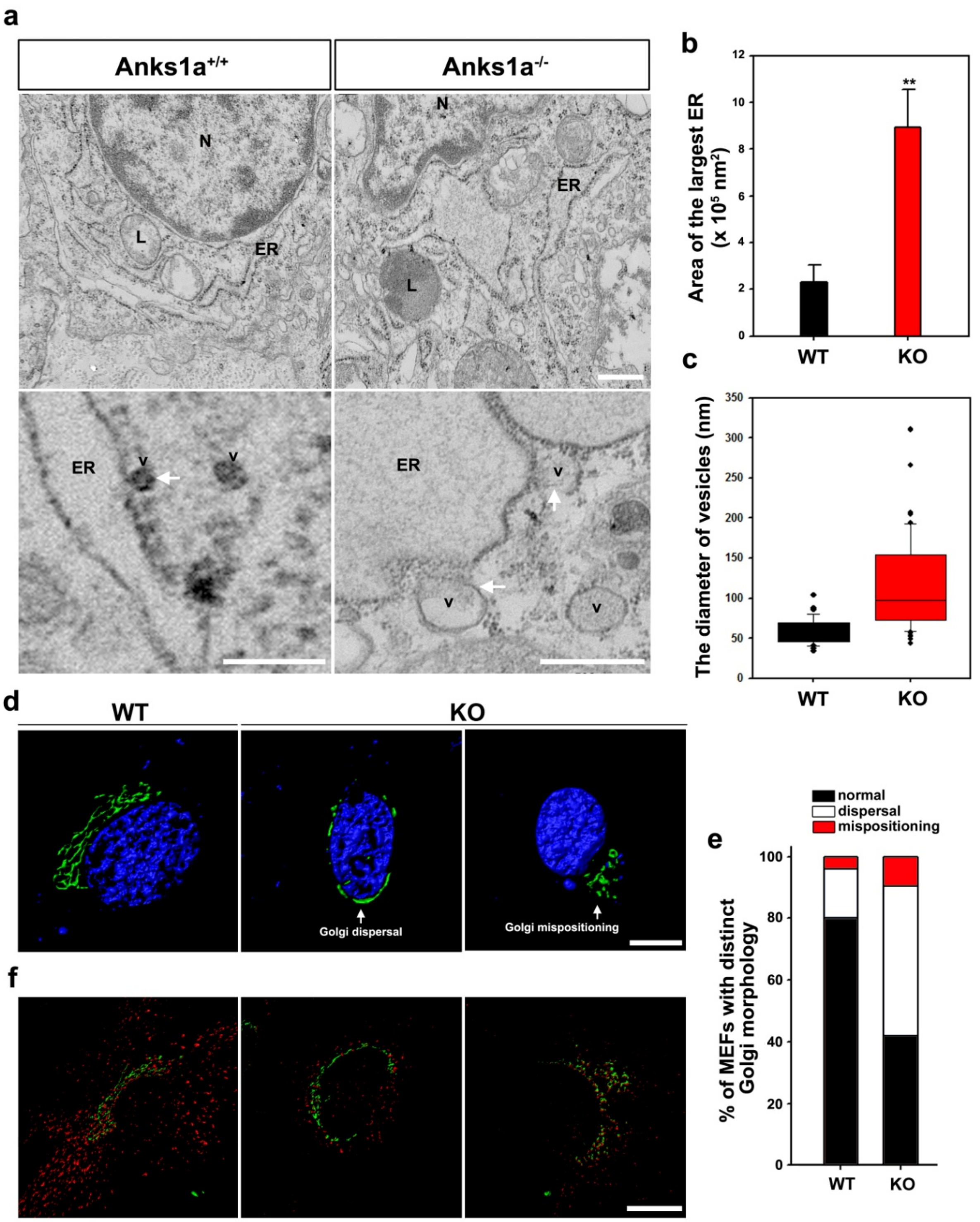

Supplementary Figure 4. Anks1a KO MEF cells show endomembrane system defects. (a) TEM analyses of MEF cells. Anks1a KO MEF cells show enlarged ERs (top panels). The vesicles budding from the ER were enlarged to the same magnification (bottom panels). N, nucleus; L, lysosome; v, vesicle. Scale bar, 500 μm. (b) The largest longitudinal ER segment

within each cell was selected for measuring its area (n=30 for WT and KO). ** $P < 0.01$, student's *t*-test. (c) The sizes of transport vesicles attached to the ER were measured using ImageJ (n=40). $P < 0.05$, student's *t*-test. (d-f) WT and Anks1a KO MEF cells were immunostained with anti-GM130 (Golgi marker) or LAMP1 (lysosome marker) antibodies. The percentage of each cell type with normal, dispersed (punctate fragments found throughout the cytoplasm), or mispositioned (far from the microtubule-organizing center) Golgi phenotypes are indicated (n=25~30 for each group). Scale bar, 10 μm.

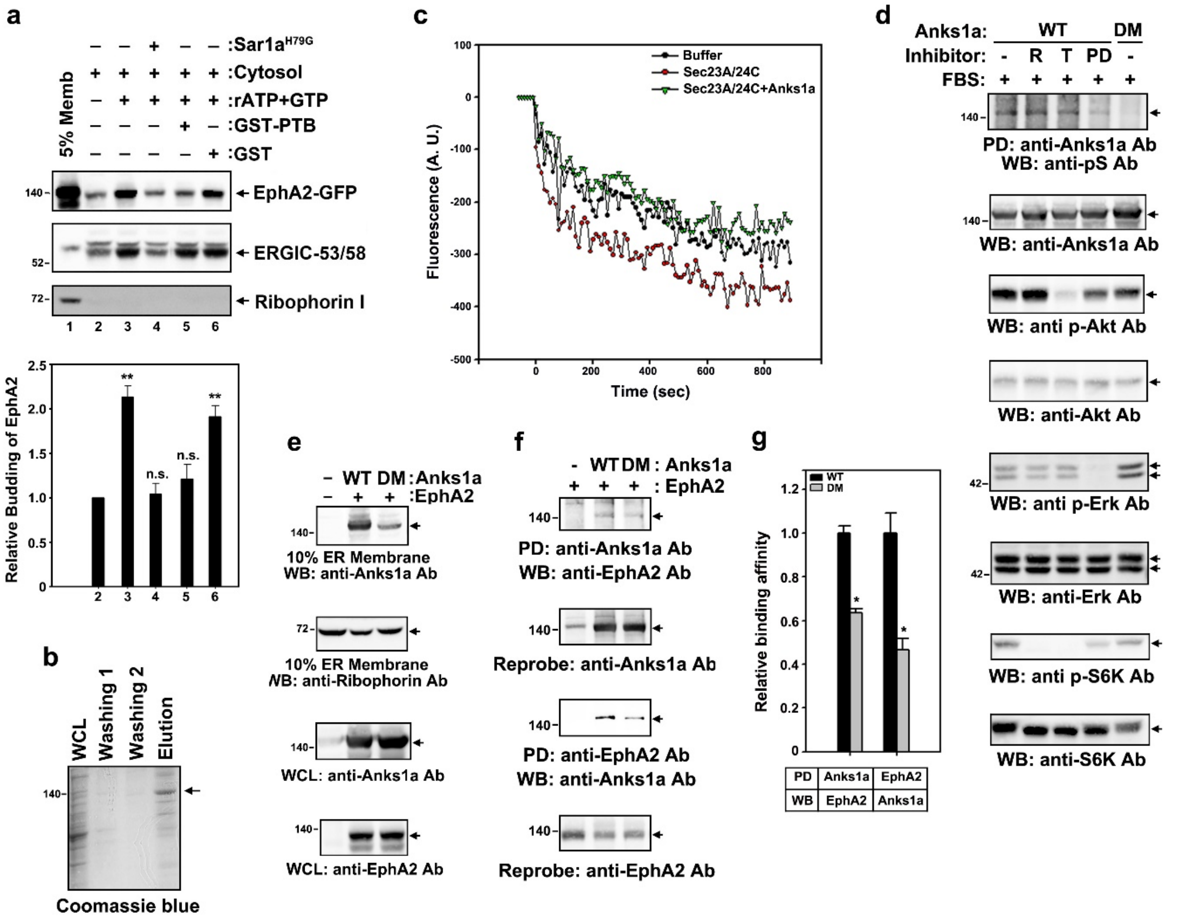

Supplementary Figure 5. Regulation of Anks1a-mediated COPII biogenesis by the PTB domain and serine phosphorylation. (a) Experiments were performed as in Fig 2f, except for the addition of the purified GST-PTB fusion protein to the budding assay. * $P < 0.05$, student's *t*-test. (b) Sf9 cells were infected with baculovirus encoding human Anks1a (tagged with His). Full-length Anks1a was purified using a Ni-NTA affinity column and stained with Coomassie blue. (c) Sar1-GTPase activity assessed by measuring Trp fluorescence. Liposomes were pre-loaded with Sar1-GTP and incubated with Sec23/24 plus or minus purified Anks1a. Then, the intrinsic Trp fluorescence of Sar1 was measured because Trp fluorescence falls as Sar1-GTP is converted to Sar1-GDP. Note that the presence of Anks1a abolishes the GAP activity of Sec23/24. The results presented in this panel were reproducible in at least three independent experiments. (d) Experiments were performed as described in Fig. 3b and c, except that cells were treated for 1 hour with the indicated chemicals before serum stimulation. PD, PD98059; R, rapamycin; T, torin. (e) Experiments were performed as in Supplementary Fig. 3h, except for the use of the Anks1a-DM mutant. (f) Experiments were performed as in Fig. 3b and c, except that cell lysates were subjected to reciprocal immunoprecipitations with the indicated antibodies. (g) The data presented in Supplementary Fig. 5f were quantified based on three independent experiments. * $P < 0.05$, student's *t*-test.

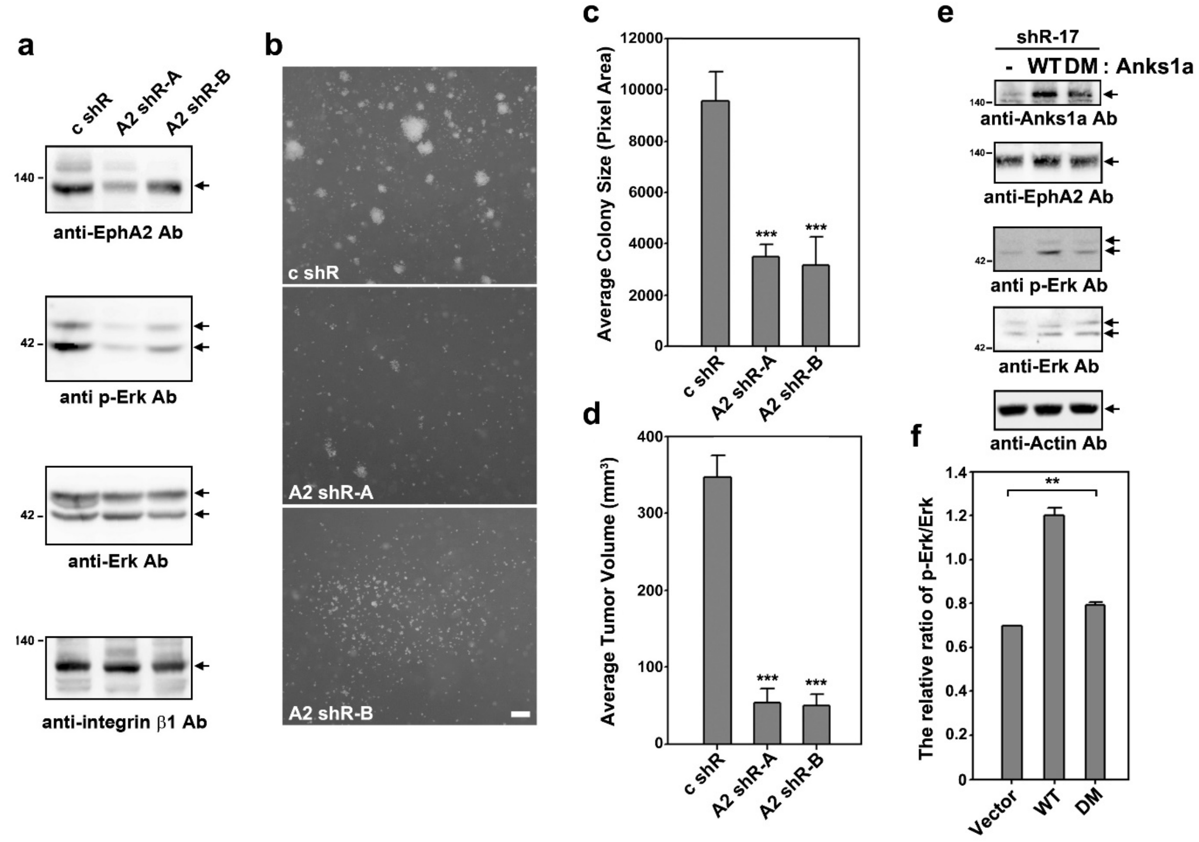

Supplementary Figure 6. Down-regulation of EphA2 or Anks1a reduces the tumorigenic growth of CT26 cells. (a-d) Experiments were performed essentially as described in Fig. 4, except that CT26 cells were infected with EphA2-specific shRNA lentiviruses. Scale bar, 50 μm. *** $P < 0.001$, student's *t*-test. (e and f) Experiments were performed as described in Fig. 4, except that Anks1a-specific shRNA17 infected cells were further transfected with the indicated constructs expressing human wild type Anks1a or the Anks1a Ser DM mutant. Note that these human Anks1a DNAs are resistant to mouse Anks1a-specific shRNAs. ** $P < 0.01$, one-way ANOVA test.

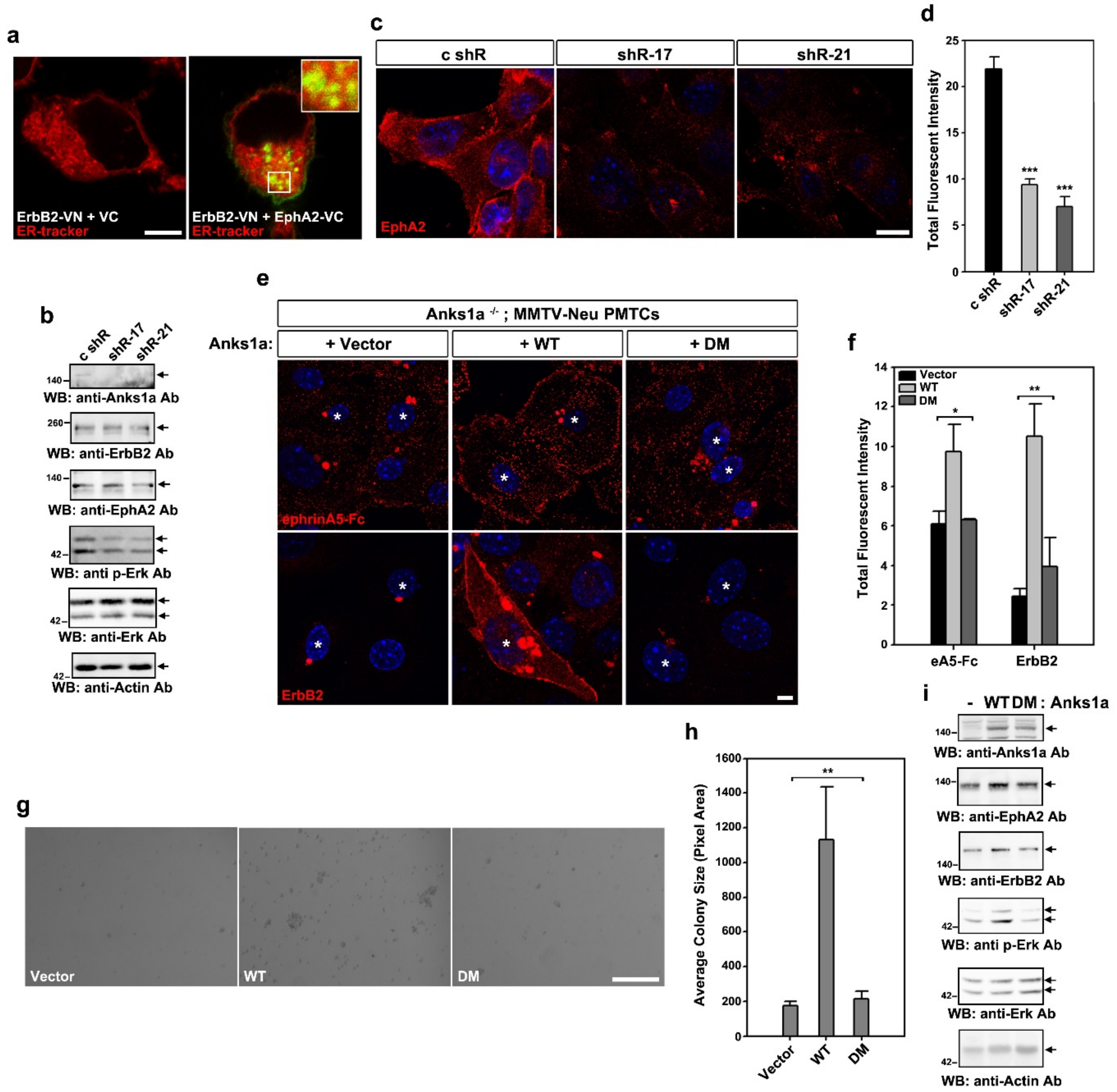

Supplementary Figure 7. The interaction between Anks1a and EphA2 is important for ErbB2-mediated tumorigenesis. (a) Experiments were performed as described in Fig. 1g, except for the substitution of the indicated constructs. Scale bar, 10 μm. (b) Cell lysates from PMTCs infected with each Anks1a-specific shRNA lentivirus were immunoblotted with the indicated antibodies. The arrows mark the proteins recognized by each antibody. (c and d) Experiments were performed as described in Fig. 5d and e, except for the substitution of anti-EphA2 antibodies. Scale bar, 20 μm. *** $P < 0.001$, student's t-test. (e and f) *Anks1a*$^{-/-}$*; MMTV-Neu* PMTCs were cultured and then transfected with the indicated constructs. Then, immunofluorescent staining was performed as described in Fig. 5d and e and the transfected cells were visualized with GFP. These are marked with asterisks. Note that the Anks1a-Ser DM mutant does not rescue the cell surface localization of EphA2 or ErbB2 in *Anks1a*$^{-/-}$*; MMTV-Neu* PMTCs. Scale bar, 10 μm. * $P < 0.05$; ** $P < 0.01$, one-way ANOVA tests. (g and h) Experiments were performed as described in Fig. 5b and c. Colonies arising from the vector or Anks1a-Ser DM transfected *Anks1a*$^{-/-}$*; MMTV-Neu* PMTCs were barely detectable until five weeks. Scale bar, 250 μm. (i) Experiments were performed as described in

Supplementary Fig. 7b, except that *Anks1a*$^{-/-}$*; MMTV-Neu* PMTCs were transfected with the indicated constructs.

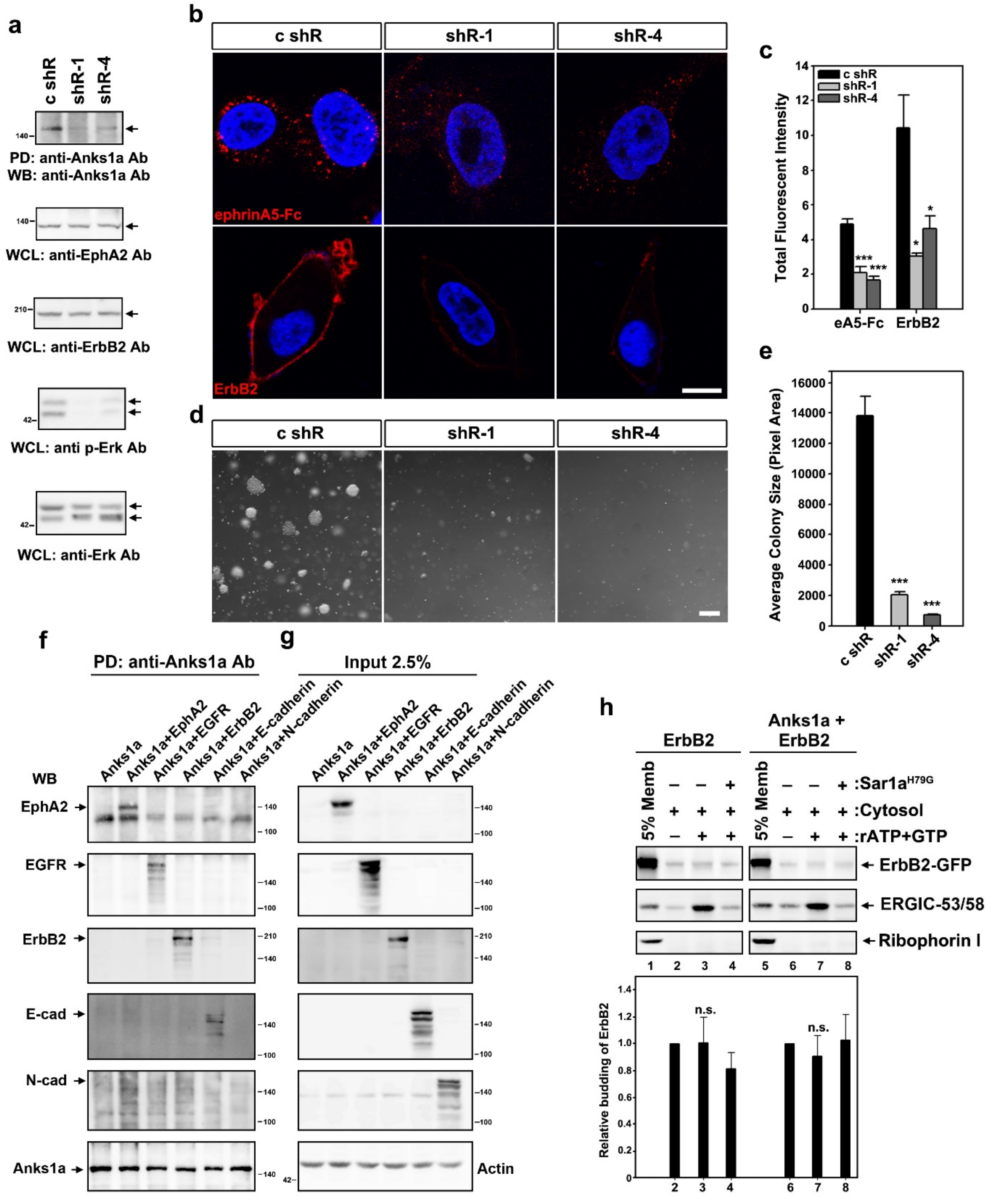

Supplementary Figure 8. The role of Anks1a in the EphA2-mediated ErbB2 signaling. (a) Experiments were performed as described in Fig. 4a, except that the SK-BR-3 human breast cancer cell line was infected with human-specific shRNA lentiviruses. Note that Anks1a depletion significantly reduces Erk signaling. (b and c) Experiments were performed as described in Fig. 5d and e, except that human Anks1a was down-regulated in SK-BR-3 human cells. Scale bar, 10 μm. *** $P < 0.001$, student's t-test. (d and e) Experiments were performed as described in Fig. 5b and c, except that human Anks1a was down-regulated in

SK-BR-3 human cells. Scale bar, 250 μm. *** $P < 0.001$, student's *t*-test. (f and g) Experiments were performed as in Fig. 3b and c, except that cell lysates were subjected to immunoprecipitation with anti-Anks1a antibodies and immunoblotted with the indicated antibodies. (h) Experiments were performed as described in Fig. 5f.

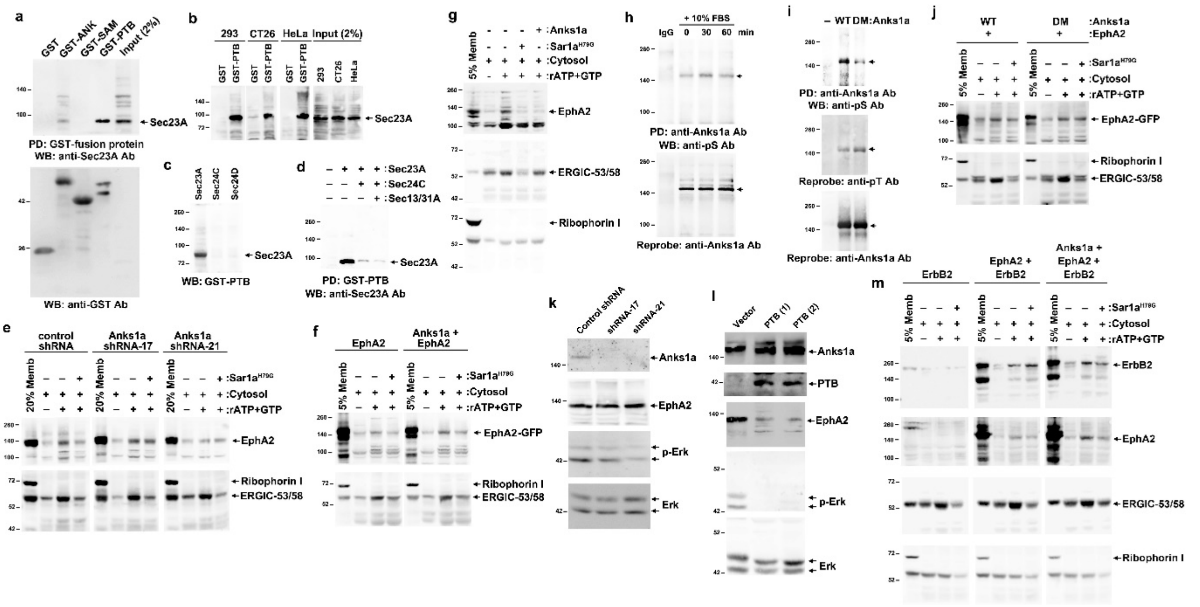

Supplementary Figure 9. Uncropped images for main figures. (a-f) Uncropped images for Fig. 2. (g-j) Uncropped images for Fig. 3a-d. (k and l) Uncropped images for Fig. 4a and g. (m) Uncropped images for Fig. 5f.
